# The effects of the arts and arts therapies on mental health outcomes of children and young people: a systematic review and meta-analysis

**DOI:** 10.1016/j.eclinm.2026.104261

**Published:** 2026-10-15

**Authors:** Pui Sze Cheung, Sarah Rhodes, Elisabeth Bahr, Wilfried Lakena, Nisha Sajnani, Nils Fietje, Vicky Karkou

**Affiliations:** a Faculty of Health, Social Care and Medicine, Research Centre for Arts and Wellbeing, Health Research Institute, Edge Hill University, Saint Helens Road, Ormskirk, Lancashire, L39 4QP, UK; b Faculty of Biology, Medicine and Health, Centre for Biostatistics, School of Health Sciences, The University of Manchester, Oxford Road, Manchester, M13 9PL, UK; c Jameel Arts & Health Lab, NYU Steinhardt, New York University, 80 Washington Square East, New York, NY, 10003, USA; d Behavioural and Cultural Insights Team, WHO Regional Office for Europe, Copenhagen, Denmark

**Keywords:** Mental health, Paediatrics, Psychiatry, Arts therapies, Arts-based intervention, Meta-analysis

## Abstract

**Background:**

There is growing evidence on the positive effects of the arts and arts therapies for mental health (MH) outcomes. This review aims to fill a research gap by establishing the evidence on the effects of arts-based interventions on selected MH outcomes for children and young people (CYP).

**Methods:**

Our systematic review and meta-analysis included randomised controlled trials with CYP aged 5–24 years receiving any type of arts-based intervention. Five databases (CINAHL, Embase.com, MEDLINE, PsycINFO, and Web of Science) were searched for studies published between 1/1/2005 and 18/5/2025. Two reviewers independently screened all records and assessed risk-of-bias using Cochrane Risk of Bias 2 (RoB2) and certainty using Grading of Recommendations Assessment, Development, and Evaluation (GRADE). Data were initially extracted using the AI-assisted review tool Elicit and independently verified by two reviewers. All data were pooled using random-effects meta-analysis, and effect estimates for MH outcomes are summarised as Standardised Mean Differences (SMD) with 95% confidence intervals (CI). Heterogeneity was assessed using the *I*^*2*^ statistic, with pre-specified subgroup and sensitivity analyses conducted to explore possible sources of inconsistency. This study is registered with PROSPERO (CRD420251015352).

**Findings:**

One hundred trials across 29 countries involving CYP aged 5–24 years with a range of health conditions, alongside healthy populations, were included in this review. Based on studies with some concerns or high risk-of-bias, arts-based interventions were found to reduce individuals' depression (1.06 SMD lower [95% CI 0.45–1.67, n = 1655, *I*^*2*^ = 97%]), anxiety (0.75 SMD lower [95% CI 0.57–0.94, n = 3898, *I*^*2*^ = 87%]), and increase individuals’ wellbeing (0.53 SMD higher [95% CI 0.26–0.80, n = 538, *I*^*2*^ = 49%]) and quality of life (0.54 SMD higher [95% CI 0.25–0.83, n = 441, *I*^*2*^ = 53%]) on average post-intervention. Certainty of results relating to wellbeing and quality of life was low due to imprecision and concerns about risk of bias, and very low for depression and anxiety due to additional inconsistency associated with high heterogeneity. Findings relating to anxiety were further supported by biomarker data (blood pressure and heart rate) with moderate certainty.

**Interpretation:**

This systematic review provides a comprehensive synthesis of evidence from randomised controlled trials assessing the effects of a wide variety arts-based interventions on the mental health of CYP. Although the strength of any conclusions is limited by heterogeneity, imprecision and bias of the studies assessed, average effects were similar to those typically reported for established non-arts-based psychosocial interventions, including talking therapies. These findings advance our understanding of arts-based therapeutic strategies for CYP's MH. Further high-quality trials, with larger sample sizes and standardised outcome measures and timepoints, are needed to improve the certainty of evidence in this area.

**Funding:**

10.13039/100014013UKRI.

**Research in context**

**Evidence before this study**

Mental health problems among children and young people (CYP) are increasingly common, while existing talking-therapy–based services provide only small to moderate effects and are often difficult to access. Growing evidence indicates that arts-based interventions can have a positive impact, but a comprehensive synthesis of evidence relating to CYP remains unclear. Prior to the current systematic review and meta-analysis, we conducted an umbrella review of arts-based interventions on Non-Communicable Diseases (NCDs). We searched The Cochrane Database of Systematic Reviews, Web of Science, Embase, MEDLINE, CINAHL and PsycINFO from database inception to 7 September 2023, using terms relating to arts-based interventions (art, drama, music, dance, poetry, digital arts, and film) and study design (systematic review, meta-analysis) and screened for NCDs (mental health conditions, cancer, CVD, CRD, diabetes and neurological conditions). The search yielded 3885 unique citations. Of the 150 eligible systematic reviews, 30 (78 effect sizes) related to mental health conditions, revealing medium overall effects (0.53, 95% Confidence Interval (CI) 0.39–0.67) including medium-to-large effects for anxiety/stress (0.71, 95% CI 0.46–0.97, 11 effect sizes), and medium effects for depression (0.57, 95% CI 0.42–0.73, 24 effect sizes) and mood (0.68, 95% CI 0.09–1.26, 1 effect size). Using the updated MeaSurement Tool to Assess systematic Reviews (AMSTAR-2), one third of the systematic reviews included in this study were rated as having moderate to high methodological quality ratings, a comparable level of quality with other related fields. However, there were not sufficient reviews addressing CYP's mental health, highlighting a clear need to synthesise evidence at a primary trial level for this age group.

**Added value of this study**

To the best of our knowledge, this is the first time that there is a comprehensive meta-analysis of RCTs examining the effects of all types of arts-based interventions on mental health outcomes related to CYP and the first comprehensive synthesis of evidence that combines both psychological outcomes and relevant physiological biomarkers. We find that arts-based interventions reduce depression and anxiety, improve wellbeing and quality of life, and have positive effects on heart rate and blood pressure. Biomarker data and subgroup analysis supported self-reported outcomes. However, limitations, such as high heterogeneity and small sample sizes should be considered when interpreting the findings.

**Implications of all the available evidence**

Our findings support further discussion on the role of arts-based interventions as accessible and effective mental health support options for CYP, with average effects broadly similar to those reported for established talking therapies. Further high-quality trials, with larger sample sizes and standardised outcome measures and timepoints, and incorporating physiological biomarkers alongside mental health outcomes, are needed to improve the certainty of evidence in this area.

## Introduction^e^

Due to an increase in prevalence, the mental health (MH) of children and young people (CYP) has received renewed attention. World Health Organisation (WHO)^1^ reports that one in seven CYP globally are faced with MH concerns. Similarly, the Organisation for Economic Co-operation and Development (OECD)^2^ reports that from 2012 to 2022 youth MH worsened in nine out of the 11 countries analysed, with the average annual decline in mental health indicators ranging from approximately 3%–16%, depending on the country. Modern stressors may contribute to the increase in poor mental health. The OECD^2^ refers to digitalisation and broader social, economic, and environmental changes as key risk factors alongside long-standing issues such as inequalities, poverty, academic pressures, and bullying. These modern stressors are reflected in studies around the world; in India for example, links have been made between decreased MH and extensive and/or inappropriate use of social media.^3^

Services are also often unavailable for CYP with MH needs, in some cases with waiting lists of two or more years.^4^ When services become available, they are often limited to talking therapies with Cognitive Behavioural Therapy (CBT) being the main treatment.^5^ However, it is imperative that services are optimised through comparative research,^6^ increasing effective treatment options that meet diverse CYP needs.

The arts and arts therapies may benefit CYP by bridging the gap between need and care, offering an additional way of addressing MH needs. In this article, arts-based interventions cover a broad range of creative activities, including dance, literary arts, music, visual art, theatre, and media, while arts therapies specifically refer to interventions that involve a therapeutic relationship within an agreed therapeutic frame, delivered by a qualified arts therapist. CYP often indicate benefits from these types of interventions,^7^ with studies suggesting low dropout rates and high acceptability.^8^^,^^9^ For example, in a school-based study that used arts therapies, children discussed opportunities to articulate their feelings through the use of creative media, while others mentioned emotional regulation.^7^ However, the utilisation of arts-based interventions as a preventive or therapeutic approach for MH in CYP remains limited, likely due to the perception that such interventions lack a robust evidence base. Existing reviews of evidence tend to be either broad scoping reviews or too specific. For instance, the 2019 WHO scoping review^10^ synthesised more than 3000 studies on the role of the arts in health for well-being. In contrast, Cochrane reviews often examine specific artistic forms (e.g., dance, music therapy).^11^^,^^12^ Recent literature however, argues that there are common therapeutic factors across the arts impacting on MH outcomes^13^ and underpinning physiological^14^ and psychological^15^ mechanisms of change across arts engagement. Further, an umbrella review completed by this team regarding non-communicable diseases^16^ did not detect differences between different art forms or disciplines within the wider arts and health and arts therapies fields. Still, most reviews that examine arts-based interventions for CYP focus on either arts therapies^17^, ^18^, ^19^ or participatory arts activities.^20^^,^^21^ A comprehensive synthesis of evidence from the broader field of arts and health, specifically addressing CYP, remains unavailable.

To address this gap, this review synthesises evidence on MH outcomes across all types of arts-based interventions for CYP aged 5–24, drawing exclusively from randomised controlled trials (RCTs). It examines the effects of these interventions on depression, anxiety, wellbeing, and health-related quality of life (hereafter referred to simply as ‘quality of life’–QoL), and physiological biomarkers including heart rate, blood pressure and cortisol. This review also explores consistency of effects across vulnerable and healthy populations, different age groups, arts modalities, engagement types, and intervention types, with outcomes assessed primarily immediately post-intervention (T1) and first available follow-up at least one month post intervention (T2) (secondary).

## Methods

This systematic review and meta-analysis were reported according to PRISMA guidelines. The protocol was prepared in advance and prospectively registered with the International Prospective Register of Systematic Reviews (PROSPERO; CRD420251015352), where the full study protocol is publicly available.

### PICOS (population, intervention, comparator, outcome, study design) criteria for inclusion/exclusion

We included studies with participants aged 5–24 years old and all types of arts-based intervention ranging from clinical arts therapy to non-clinical arts participation, covering both active engagement (e.g., songwriting, dancing, and art-making) and receptive engagement, where participants observed or received arts-based content (e.g., listening to music/story or watching a performance). Modalities included: (i) dance/movement, (ii) literary arts, (iii) media, (iv) music, (v) theatre/performance, and (vi) visual arts, craft, and design, or a combination of more than one art form and/or a mixed modality referred to here as ‘multimodal’. Studies were excluded if they involved broader activities outside the scope of our definition of arts such as architecture or gardening, or if they aimed to improve skills, knowledge, performance or cognitive outcomes.

RCTs and cluster-randomised trials comparing arts-based interventions with placebo, usual care, or an active control were included. Studies had to report outcomes using validated instruments for at least one of the key MH measures (depression, anxiety, wellbeing, or QoL) or relevant physiological biomarkers (heart rate, blood pressure, or cortisol). Studies conducted in any setting or country and reported in any language were included.

### Search methods for identification of studies

Systematic searches were conducted across five databases, including CINAHL, Embase.com, MEDLINE, and PsycINFO, and Web of Science Core Collection, for studies published between 1/1/2005 and 18/5/2025. Supplementary searches included Google Scholar, published systematic reviews, the Cochrane Library, and forward citation tracking. The search string included a set of terms related to the arts, such as ‘art’, ‘music’, ‘dance’, or ‘drama’; children and young people, and terms associated with ‘randomised controlled trials’. Our search strategy can be found in Supplementary Table S1.

A top-up search performed on 30/4/2026 identified 18 additional eligible trials. These studies were not incorporated into the current synthesis and analysis. A list of these studies is provided in the Supplementary Table S8.

### Study selection, data extraction, and risk-of-bias

Studies were selected using the Cochrane systematic review software Covidence (www.covidence.org).^22^ After removal of duplicates, all titles, abstracts, and full texts were independently screened by pairs of reviewers (PSC, SR, VK, and EB), with disagreements resolved through discussion.

Initial data extraction was carried out using AI-assisted systematic review tool Elicit (https://elicit.com/)^23^ based on predefined extraction criteria operationalised as structured fields. The full set of column definitions and details on AI-assisted extraction are provided in the Supplementary Table S5. This was followed by manual categorisation and extraction of summary statistics for meta-analysis and any missing details by two reviewers (PSC, SR).

All eligible studies were included in the qualitative synthesis. Studies providing sufficient quantitative outcome data were included in the meta-analysis. Risk-of-bias was assessed using the Cochrane Risk of Bias 2 (RoB2) tool for studies contributing to the meta-analysis (n = 77).^24^ Each study was assessed by one reviewer and checked by a second reviewer, with reviewer combinations drawn from PSC, SR, WL, and EB. Any discrepancies were resolved through discussion. Funnel plots were produced and assessed for asymmetry to check for publication bias. Egger's test was not performed due to the fact that several outcomes were informed by fewer than ten studies. Grading of Recommendations Assessment, Development and Evaluation (GRADE) was used to assess certainty of evidence for all the outcome measures included in this study for T1 only.

### Statistical analysis

Study and participant characteristics were summarised descriptively. Categorical variables (e.g., country, arts modality, engagement type, and comparator type) were reported as frequencies. Continuous variables (e.g., participant age, number of sessions) were reported as medians with interquartile ranges (IQRs).

We extracted means and standard deviations (SDs) at T1 and T2, where T1 was defined as end-of-intervention (assessed immediately following completion of the intervention) and T2 as follow-up (assessed at least one month after intervention completion). Standardised mean differences (SMDs) using Hedges’ *g*^25^ were used to combine data from outcomes measured using a mixture of different tools and mean differences were used for outcomes where all data were measured on the same scale (blood pressure, heart rate). Considering the clinical and methodological heterogeneity across included studies, all data were pooled using random-effects meta-analysis. The *I*^2^ statistic was reported for all analyses to describe heterogeneity. Where a study reported data on more than one arts-based intervention, the control arm was shared between intervention arms. Missing means and SDs were estimated where possible using medians and IQRs or baseline data and an effective sample size adjustment was made for a cluster-randomised trial.^26^

Prespecified subgroup analyses, using Cochran's Q-based test of subgroup differences (Q_b), were performed at T1 only to explore variation in effect size by population type (vulnerable vs healthy), age group, arts modality, arts therapy status, engagement type (active vs receptive), and comparator type (active vs passive control). Two further subgroup analyses by dose and risk-of-bias were added post-hoc, based on peer-reviewers’ suggestions during revision (see Differences from the registered protocol). 95% confidence intervals and a 5% level of significance were used throughout.

Two post-hoc sensitivity analyses were conducted, one where studies with unusually high SMDs (greater than 3) were removed (due to concerns about plausibility) (these relate depression and anxiety only) and one where studies rated as ‘high risk-of-bias’ were removed. All analyses were conducted using Stata 19.0.^27^

### Differences from the registered protocol

The outcome measures presented in this paper are a subset of those in the registered protocol,^28^ focussing on the MH outcomes. QoL was included as a primary outcome. Biomarkers were added as objective measurements to supplement standardised mental health tools. Although we aimed to extract data at three points after the end of the intervention, the general scarcity of follow-up data at six months we limited analysis to two points. We focussed on T1 as our primary time-point and considered T2 to be secondary, where available. During the subgroup analysis, to ensure clearer interpretation, all participants with any identified health-related need were combined into a single ‘vulnerable group’ including CYP experiencing depression, anxiety and other MH problems, neurodivergent populations (e.g., Autism Spectrum Disorder (ASD), Attention Deficit Hyperactivity Disorder (ADHD)) and cerebral palsy, CYP with chronic pain, cancer, those hospitalised, before, during or after surgery, other medical procedures, or dental treatment. This group was contrasted with participants with no apparent needs labelled ‘healthy population’. Additional post-hoc subgroup analyses relating to dose (number of sessions) and risk of bias were added based on suggestions from peer reviewers. Finally, although we intended to use GRADE to assess certainty of primary outcomes only, we decided to use GRADE for both primary and secondary outcomes. Full details of the study subgroup definitions and GRADE assessment are provided in the Supplementary Tables S2 and S4.

### Patient and public involvement

Patients and members of the public were not involved in the design, conduct, or reporting of this systematic review and meta-analysis. The study used previously published data and did not include direct engagement with patients or the public. Plans for dissemination are focused on academic and professional audiences.

### Ethics

Ethical approval was not required for this study as it is a systematic review and meta-analysis based on previously published data. As this study involved no primary data collection or direct participant contact, the requirement for informed consent did not apply.

### Role of the funding source

The funder had no role in the design, conduct, data collection, analysis, interpretation, or writing of the manuscript.

## Results

A literature search on 18 May 2025 identified a total of 4858 unique records. After removing duplicates, 2446 records were screened, with 2068 excluded based on title and abstract. Fifty-six full texts were identified through supplementary searches. A total of 434 full-text reports were assessed for eligibility, 334 were excluded for reasons including ineligible population, intervention, article type, study design, or comparator, lack of targeted outcome reporting or overlap in participant samples with another included study (Supplementary Table S7). A total of 102 reports were eligible; there were four reports referring to two studies (two for each study) and were merged, resulting in 100 unique studies included in the systematic review and qualitative synthesis (Supplementary Table S6).^29^, ^30^, ^31^, ^32^, ^33^, ^34^, ^35^, ^36^, ^37^, ^38^, ^39^, ^40^, ^41^, ^42^, ^43^, ^44^, ^45^, ^46^, ^47^, ^48^, ^49^, ^50^, ^51^, ^52^, ^53^, ^54^, ^55^, ^56^, ^57^, ^58^, ^59^, ^60^, ^61^, ^62^, ^63^, ^64^, ^65^, ^66^, ^67^, ^68^^,^^69^, ^70^, ^71^, ^72^, ^73^, ^74^, ^75^, ^76^, ^77^, ^78^, ^79^, ^80^, ^81^, ^82^, ^83^, ^84^, ^85^, ^86^, ^87^, ^88^, ^89^, ^90^, ^91^, ^92^, ^93^, ^94^, ^95^, ^96^^,^^97^, ^98^, ^99^, ^100^, ^101^, ^102^, ^103^, ^104^, ^105^, ^106^, ^107^, ^108^, ^109^, ^110^, ^111^, ^112^, ^113^, ^114^, ^115^, ^116^, ^117^, ^118^, ^119^, ^120^, ^121^, ^122^, ^123^, ^124^, ^125^, ^126^, ^127^, ^128^, ^129^, ^130^ Of these, 77 studies provided sufficient quantitative outcome data for inclusion in at least one meta-analysis. The study selection process is illustrated in the PRISMA flow diagram shown in Fig. 1.

**Fig. 1:**
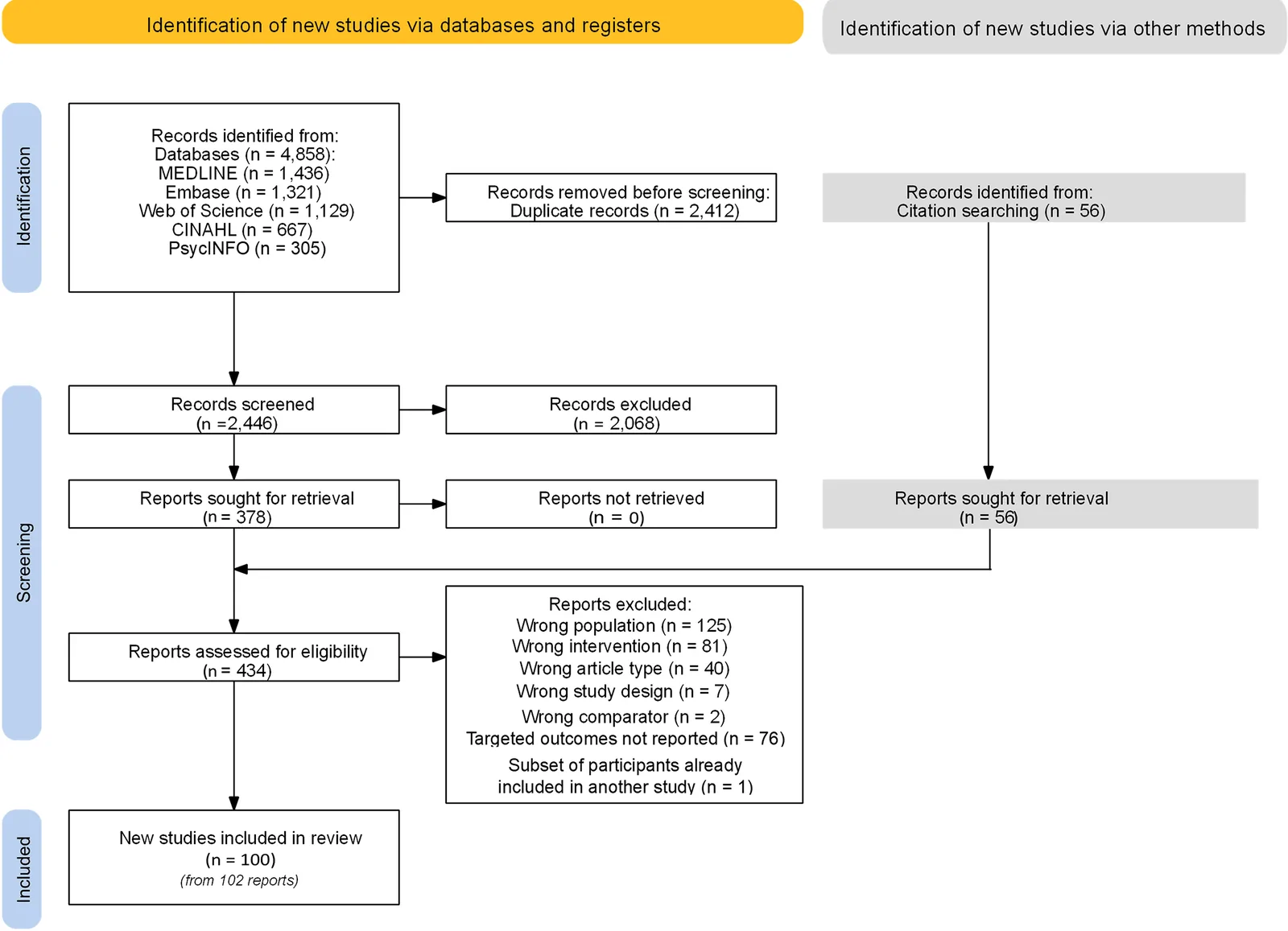
**Study profile.** Note: A top-up search conducted on 30 April 2026 identified 18 additional eligible trials, which were not incorporated into the current synthesis. PRISMA, Preferred Reporting Items for Systematic Reviews and Meta-Analyses.

The characteristics of the included studies are summarised in Table 1. Included studies were conducted across 29 countries spanning Asia, the Middle East, Europe, North and South America, Africa, and Oceania. Türkiye contributed the most studies (n = 16), followed by the Islamic Republic of Iran (n = 14), the USA and India (n = 9 each), Australia and China (n = 6 each), Republic of Korea (n = 5), and the United Kingdom (n = 4). All remaining countries contributed three or fewer studies. Most studies involved older children (9–13 years; n = 42) and young adults (18–24 years; n = 27), with smaller proportions targeting younger children (5–8 years; n = 20) and adolescents (14–17 years; n = 11). Reported mean ages ranged from 5.9 to 22.8 years. Sex distribution varied substantially across studies, ranging from all-male to all-female samples. Several paediatric neurodevelopmental studies were predominantly male, whereas many university-based studies were predominantly female. Eighty-five studies recruited participants with vulnerabilities, including those experiencing or at risk of MH problems, undergoing medical or dental procedures, or living with chronic physical conditions such as Type 1 diabetes or neurodevelopmental conditions such as ASD. Fifteen involved healthy samples with no known health needs. Study settings were predominantly clinical or educational, with 55 studies based in hospital or clinical settings and 37 in school or university environments, alongside smaller numbers in community or online settings. Sample sizes ranged from 12 participants^77^ to 463.^127^

**Table 1:** Summary of included studies (n = 100).

Study label	Country	Setting	N randomised (Arms)	Population		Intervention						Comparator	Outcomes included in analysis (Timepoint)
				Condition	Age Group	Modality	Arts therapy	Engagement	Name	Description	Dosage		
Abbasi, 2021^29^	Pakistan	Hospital/Clinical	160 (4 arms)	Vulnerable group (Dental treatment)	Younger children (5–8)	Media	No	Receptive	Dental Video Songs	Watching dental video songs on YouTube	Single session, before dental treatment	Passive control (No treatment)	HR (End of intervention)
Abd-Elshafy, 2015^30^	Egypt	Hospital/Clinical	50 (2 arms)	Vulnerable group (Cardiac surgery)	Younger children (5–8)	Music	No	Receptive	Music Listening	Listening to recorded music and songs preferred by the child	Single session, from before induction of anaesthesia until extubation	Passive control (Listening to blank CD)	SBP, Cortisol, HR (End of intervention)
Abdi, 2025^106^	Islamic Republic of Iran	Hospital/Clinical	75 (3 arms)	Vulnerable group (Hospitalisation)	Younger children (5–8)	Literary arts	No	Receptive	Storytelling	Reading age-appropriate hospital-themed books	2 sessions (1 book per day across 2 days)	Passive control (Usual care)	Not included: Measured anxiety (SCAS) but did not report summary data by group
Abdulah, 2018^107^	Iraq	Hospital/Clinical	60 (2 arms)	Vulnerable group (Cancer)	Older children (9–13)	Visual Arts, Craft and Design	No	Active	Painting and handcrafting workshops	Creating paintings and handcrafted works using watercolours, markers, wood, and cardboard	5 x 2-h sessions/week, for a month (20 x 2-h session in total)	Passive control (Usual care)	Not included: QOL (KIDSCREEN-10/HRQOL) reported as domains only
Agres, 2025^31^	Singapore	School/Educational	76 (2 arms)	Healthy population	Young adults (18–24)	Multimodal	No	Active	Movin' and Groovin' for Wellness	Facilitated drumming and dancing sessions with an emphasis on creative expression through improvisation	10 weekly sessions, 1.5 h per session	Passive control (Usual care)	Anxiety (DASS-21), Depression (DASS-21), QoL (WHOQOL-BREF) (End of intervention)
Akard, 2021^108^	USA	Online	150 (2 arms)	Vulnerable group (Cancer)	Older children (9–13)	Multimodal	No	Active	Legacy Intervention	Children created legacy projects (e.g., memory books, letters) to share with family members	One-off, completed at own pace.	Passive control (Waitlist)	Not included: QOL (PedQL) reported as domains only
Alemdar, 2023^32^	United Kingdom	Hospital/Clinical	99 (3 arms)	Vulnerable group (Hospitalisation)	Adolescents (14–17)	Music	No	Receptive	Music Listening	Listening to classical music selected by a classical music therapist	Single session, 20 min, starting 10 min before a painful procedure	Passive control (Usual care)	Cortisol, HR (End of intervention)
Alkan, 2022^33^	Türkiye	Hospital/Clinical	20 (2 arms)	Vulnerable group (Cerebral Palsy)	Older children (9–13)	Visual Arts, Craft and Design	No	Active	Photography training	Theoretical and practical training in photography to increase communication, socialisation, creativity, and wellbeing	8 weeks, 2 days a week, 120 min theoretical and 90 min application per day for the first 5 weeks, followed by 3 photo trips	Passive control (Usual care)	Anxiety (SCARED), Depression (CDI), QoL (HRQOL (KINDL)) (End of intervention)
Bali, 2025^109^	India	Hospital/Clinical	40 (2 arms)	Vulnerable group (Dental treatment)	Younger children (5–8)	Music	No	Active	Piano Music App	Child taps screen to play nursery rhymes	Single session during dental treatment	Active control (Behavioural guidance)	Not included: Anxiety (MCDAS) measured but change scores only reported
Barrera, 2018a, 2018b^110^^,^^111^	Canada	Hospital/Clinical	75 (2 arms)	Vulnerable group (Cancer–siblings)	Older children (9–13)	Music & Visual Arts, Craft and Design	No	Active	Fun with Music/Art	Group-based arts and social activities for siblings of children with cancer	8 weekly 2-hour sessions	Active control (SibCT)	Not included: Measured depression (CDI), QOL (PedsQL) but summary data not reported
Bay, 2021^112^	Chile	Hospital/Clinical	100 (2 arms)	Vulnerable group (Endoscopy)	Older children (9–13)	Music	No	Receptive	Music Listening	Children listened to self-selected music	Single session during endoscopy procedure	Passive control (Usual care)	Not included: Anxiety (m-YPAS, VAS) measured but SDs not reported
Beebe, 2010^113^	USA	Hospital/Clinical	22 (2 arms)	Vulnerable group (Asthma)	Older children (9–13)	Visual Arts, Craft and Design	Yes	Active	Art Therapy	Group art therapy sessions for children with asthma	7 weekly 1-h sessions	Passive control (Waitlist)	Not included: QOL (PedsQL) reported as change scores only
Betzalel, 2017^34^	Israel	Community	187 (3 arms)	Vulnerable group (Parental absence)	Older children (9–13)	Literary arts	Yes	Active	Bibliotherapy (superhero/affective stories)	Group bibliotherapy using superhero stories to address parental absence, trauma, fear, and anxiety	Eight 50-min sessions over ten weeks	Passive control (No treatment)	Anxiety (RCMAS-2) (End of intervention; ≥1-month follow-up)
Bhusari, 2023^35^	India	Hospital/Clinical	45 (3 arms)	Vulnerable group (Dental treatment)	Older children (9–13)	Music	No	Receptive	Binaural beats	Listening to soft, relaxing music with binaural beats (400 Hz and 412 Hz) through over-the-ear headphones	Single session, during a 45-min dental procedure	Passive control (No treatment)	Anxiety (Venham's picture) (End of intervention)
Bhusari, 2025^36^	India	Hospital/Clinical	75 (3 arms)	Vulnerable group (Dental treatment)	Older children (9–13)	Music	No	Receptive	Music of Choice	Listening to their preferred music during dental procedures	Single session, during a 45-min restorative procedure	Passive control (No treatment)	Anxiety (Venham's picture), HR (End of intervention)
Bogaert, 2024^114^	Belgium	Education	187 (2 arms)	Healthy population	Adolescents (14–17)	Literary arts	No	Active	CREAT	Creative writing via exercises using funny and thought-provoking writing prompts.	4 sessions (50 min each), weekly over 4 weeks + homework	Active control (Positive Events Training)	Not included: Measured Wellbeing (SWEMWBS) and Depression (DASS21) but unclear detail on number of clusters
Bulut, 2020^37^	Türkiye	Hospital/Clinical	140 (4 arms)	Vulnerable group (Surgery)	Younger children (5–8)	Music	No	Receptive	Music Pillow	Listening to music through Creatone music pillow to help children cope with pain and stress	Single session, 20 min every other 20 min for an hour, after returning from the postanesthesia care unit	Passive control (Usual care)	Anxiety (mYPAS) (End of intervention)
Burns, 2009^115^	USA	Hospital/Clinical	12 (2 arms)	Vulnerable group (Cancer–stem-cell transplantation)	Young adults (18–24)	Media	Yes	Active	Therapeutic Music Video (TMV)	Participants created personalised music video using song selection, lyric writing, and visual imagery	6 sessions (∼30–60 min), twice weekly over 3 weeks	Active control (Audiobook)	Not included: Anxiety (STAIC), Wellbeing (IWB), QOL (LASA) measured but only 1 participant with outcome data in intervention group. SD = 0
Chawla, 2025^38^	India	Hospital/Clinical	350 (5 arms)	Vulnerable group (Dental treatment)	Younger children (5–8)	Music	No	Receptive	Music listening	Listening to child-friendly, relaxing songs selected by the patient	Single session, during dental procedure	Active control (Gustatory distraction using honey for taste)	Anxiety (CBCS) (End of intervention)
Chen, 2015^39^	Taiwan	School/Educational	71 (2 arms)	Vulnerable group (Depressed mood)	Young adults (18–24)	Music	No	Receptive	Chinese five-element music listening	Listening to Chinese five-element music played on Chinese musical instruments, with a minor mode, slow tempo, and simple structures	40 min twice per week for 10 weeks	Passive control (Usual care)	Cortisol, Depression (DMSRIA) (End of intervention)
Chen, 2019^40^	Taiwan	School/Educational	65 (2 arms)	Healthy population	Older children (9–13)	Music	No	Active	Preferred Music Listening and Singing	Listening to and singing music as part of a therapeutic process	40 min twice per week for 10 weeks	Passive control (Usual care)	Cortisol, Depression (BDI-II) (End of intervention)
Cheng, 2023^41^	China, Hong Kong SAR	School/Educational	64 (2 arms)	Healthy population	Young adults (18–24)	Music	Yes	Active	Group-based Focus Music Imagery Program	Combines music listening with imagery techniques to promote emotional exploration and relaxation	6 weekly sessions of 120 min	Active control (Breathing and stretching exercises, guided imagery, and still-life drawing with music)	Anxiety (GAD-7) (End of intervention; ≥1-month follow-up)
Cheraghi, 2021^42^	Islamic Republic of Iran	Hospital/Clinical	120 (3 arms)	Vulnerable group (Burn dressing changes)	Younger children (5–8)	Music	No	Receptive	Music listening	Listening to children songs through headphones	Single session, 2 min before dressing change until the end of the procedure (approximately 10 min)	Passive control (Usual care)	HR (End of intervention)
Cheung, 2019^44^	China, Hong Kong SAR	Hospital/Clinical	60 (2 arms)	Vulnerable group (Brain tumour survivor)	Older children (9–13)	Music	No	Active	Musical Training	One-to-one musical training conducted by qualified orchestral performers at participants' homes	45 min, Weekly for 52 weeks	Passive control (Placebo intervention)	Depression (CES-DC), QoL (PedsQL) (End of intervention)
Choi, 2023^43^	Republic of Korea	Hospital/Clinical	22 (2 arms)	Vulnerable group (Tic disorder)	Older children (9–13)	Visual Arts, Craft and Design	No	Active	Art relaxation technique	Watercolour painting, clay exercise, colouring mandalas, and free drawing	40 ± 5 min per session, once a week for four weeks	Passive control (Waitlist control)	HR (End of intervention)
Çitil, 2024^45^	Türkiye	School/Educational	72 (2 arms)	Vulnerable group (PMS)	Young adults (18–24)	Multimodal	No	Active	Group arts session	Group sessions involving music, dance, visual arts, and literary arts activities	Weekly for 8 weeks, 30–35 min per session	Passive control (Usual care)	Anxiety (PMSS-anxiety), Depression (PMSS-depression) (End of intervention)
Cleary, 2017^116^	Australia	Education	19 (2 arms)	Vulnerable group (Cerebral palsy)	Older children (9–13)	Multimodal	No	Active	Arts and social programme	Creative activities including painting and hand sewing, cooking, music playing, and puppet making	27 sessions over 9 weeks (3 × 30-min sessions/week)	Active control (Aerobic exercise)	Not included: QOL (CP OQL-Child) reported as domains only
Corbett, 2017^46^	USA	School/Educational	30 (2 arms)	Vulnerable group (ASD)	Older children (9–13)	Theatre/Performance	No	Active	SENSE Theatre	Peer-mediated, theatre-based intervention involving role-play, character development, singing, basic choreography, and improvisation	10 weekly sessions, each lasting 4 h	Passive control (Waitlist control)	Anxiety (STAI) (End of intervention)
Corbett, 2023^47^	USA	School/Educational	290 (2 arms)	Vulnerable group (ASD)	Older children (9–13)	Theatre/Performance	No	Active	SENSE Theatre	Peer-mediated, theatre-based intervention involving role-play, character development, singing, basic choreography, and improvisation	10 sessions, approximately 3 h per session, once a week	Active control (Tackling Teenage Training program)	Anxiety (CASS (social)) (End of intervention; ≥1-month follow-up)
Currie, 2012^48^	Australia	School/Educational	65 (2 arms)	Vulnerable group (Aggressive behaviour)	Adolescents (14–17)	Music	No	Active	Doing Anger Differently	Structured Latin American percussion exercises to treat reactive aggression in adolescent males	Twenty sessions over ten weeks	Passive control (Waitlist control)	Depression (BDI) (End of intervention; ≥1-month follow-up)
De la Torre-Luque, 2017^49^	Spain	School/Educational	58 (2 arms)	Healthy population	Young adults (18–24)	Music	No	Receptive	Preferred music listening	Listening to their preferred relaxing music to aid in stress recovery	Single session lasting 15 min	Passive control (Silent resting)	Anxiety (STAI), Depression (ST-DEP), HR (End of intervention)
Dehghan, 2017^50^	Islamic Republic of Iran	Hospital/Clinical	75 (3 arms)	Vulnerable group (Appendicitis surgery)	Older children (9–13)	Theatre/Performance	No	Active	Dramatic Puppet	A researcher wearing puppet clothes engages with the child	During the morning of surgery in three stages: introduction, procedural assistance, and accompaniment to the operating room	Passive control (No treatment)	Anxiety (RCMAS), Cortisol (End of intervention)
Erkut, 2024^51^	Türkiye	Hospital/Clinical	150 (2 arms)	Vulnerable group (Medical procedure)	Older children (9–13)	Music	No	Receptive	Guided Imagery	Listening to a sound clip that guides children to imagine walking in a forest, with slow music and nature sounds	Single session, for 2.35 min	Passive control (Usual care)	HR (End of intervention)
Forouzandeh, 2020^52^	Islamic Republic of Iran	Hospital/Clinical	172 (3 arms)	Vulnerable group (Surgery)	Younger children (5–8)	Visual Arts, Craft and Design	No	Active	Painting	Drawing and colouring with provided materials	Single session, 20–30 min before surgery	Passive control (Usual care)	Anxiety (YPAS) (End of intervention)
Franzoi, 2016^117^	Brazil	Hospital/Clinical	52 (2 arms)	Vulnerable group (Pre-operative)	Younger children (5–8)	Music	No	Receptive	Music Listening	Children listened to preselected instrumental and child-friendly songs (60–80 bpm) via MP3 player	Single 15-min session preoperatively	Passive control (Usual care)	Not included: Anxiety (yPAS), HR, BP measured but SDs not reported
Gee, 2019^53^	United Kingdom	School/Educational	68 (2 arms)	Vulnerable group (Stressed, anxious, or depressed)	Young adults (18–24)	Music	No	Active	Songwriting	Students create and record two songs	5 weekly sessions, each lasting 2 h	Passive control (Waitlist control)	Depression (HADS-D) (End of intervention)
Goktas, 2023^54^	Türkiye	Hospital/Clinical	144 (4 arms)	Vulnerable group (Medical procedure)	Older children (9–13)	Music	No	Receptive	Auditory distraction	Listening to self-selected songs through over-ear wireless headphones	Approximately 3 min before, during, and after the invasive procedure	Passive control (Usual care)	Anxiety (CAM-S) (End of intervention)
Gold, 2017^55^	Australia	School/Educational	100 (2 arms) ∗Cluster-RCT	Vulnerable group (Mental health problems)	Adolescents (14–17)	Music	Yes	Active	Group Music Therapy	Group activities including improvisation, song contributions, songwriting, and playlist creation	Weekly sessions over eight weeks	Active control (Self-Directed Music Listening)	Depression (K10), WB (MHC-SF) (End of intervention)
Goldbeck, 2012^56^	Germany	Hospital/Clinical	36 (2 arms)	Vulnerable group (Anxiety disorder)	Older children (9–13)	Music	Yes	Active	Multimodal Music Therapy	Individual and group sessions with active music play and cognitive-behavioural interventions	15 sessions over a mean duration of 17.6 weeks	Passive control (Treatment as usual)	Anxiety (STAIC-T), Depression (CDI), QoL (ILQ) (End of intervention)
Gómez Scarpetta, 2012^57^	Colombia	Hospital/Clinical	30 (2 arms)	Vulnerable group (Down Syndrome, dental treatment)	Older children (9–13)	Music	No	Receptive	Music Listening	Listening to “Las cuatro estaciones de Vivaldi” in the background	Single session, from entering the dental office until 5 min after the procedure	Passive control (Usual care)	HR (End of intervention)
Gozin, 2022^118^	Islamic Republic of Iran	Hospital/Clinical	45 (3 arms)	Vulnerable group (Dental treatment)	Younger children (5–8)	Media	No	Receptive	Audio-visual Storytelling	Children watched audio-visual storytelling to reduce dental anxiety before treatment	Single session before dental treatment	Passive control (Usual care)	Not included: Anxiety (Venham's Picture Test, FIS) measured but SDs not reported
Gulmez, 2023^58^	Türkiye	School/Educational	87 (3 arms)	Healthy population	Young adults (18–24)	Music	No	Receptive	Music Listening	Listening to music in Hicaz or Huseyni maqam	30 min a day for a week	Passive control (No treatment)	Anxiety (BAI) (End of intervention)
Günay, 2022^59^	Türkiye	Hospital/Clinical	68 (2 arms)	Vulnerable group (Cancer)	Older children (9–13)	Visual Arts, Craft and Design	No	Active	Making jewellery from beads	Creating jewellery from beads with guidance	Twice a week for four weeks, with each session lasting 1.5 h	Passive control (Usual care)	Anxiety (STAI) (End of intervention)
Gurcan, 2021^60^	Türkiye	Hospital/Clinical	60 (2 arms)	Vulnerable group (Hospitalised with cancer)	Adolescents (14–17)	Visual Arts, Craft and Design	No	Active	Mandala drawing	Drawing and painting mandalas while listening to classical or instrumental music to express their feelings	1–2 h per session, two sessions with an interval of 2 or 3 days	Passive control (Usual care)	Anxiety (HADS), Depression (HADS) (End of intervention)
Hides, 2019^61^	Australia	Online/Virtual	169 (2 arms)	Vulnerable group (Mental distress)	Young adults (18–24)	Music	No	Active	Music eScape	A mobile app that guides young people to identify and manage emotions using music by analysing their music library and creating mood-based playlists	Self-directed over 1 month	Passive control (Waitlist control)	Depression (K10), WB (MHC-SF) (End of intervention)
Hosseini, 2023^119^	Islamic Republic of Iran	Education	80 (2 arms)	Vulnerable group (Dysmenorrhoea)	Young adults (18–24)	Visual Arts, Craft and Design	No	Active	Mandala Painting	Participants created mandalas to reduce anxiety, pain severity and distress of primary dysmenorrhoea	1 mandala/day, 5 days per menstrual cycle, over 2 cycles	Passive control (Usual care)	Not included: Anxiety not reported in extractable format (ranks only).
Irons, 2012^120^	Australia	Hospital/Clinical	51 (2 arms)	Vulnerable group (Cystic fibrosis)	Older children (9–13)	Music	Yes	Active	Singing Therapy	Participants engaged in structured singing exercises (posture, diaphragmatic breathing, vocal warm-ups, songs)	8 individual sessions during hospitalisation (∼2 weeks) + daily home practice post-discharge	Active control (Recreational sessions)	Not included: QOL (CFQ-R) reported as domains only
Janthasila, 2023^62^	Thailand	School/Educational	128 (4 arms)	Vulnerable group (Dental treatment)	Older children (9–13)	Music	No	Receptive	Music Listening	Listening to popular instrumental Thai pop songs	Single session, during dental treatment	Passive control (No treatment)	SBP, HR (End of intervention)
Javed, 2021^63^	Pakistan	Hospital/Clinical	76 (2 arms)	Vulnerable group (Medical procedure)	Older children (9–13)	Theatre/Performance	No	Active	Medical Clowning with Soap Bubbles	Interaction with a medical student clown and soap bubbles	Single session, during IV catheter placement	Passive control (Usual care)	Anxiety (STAI) (End of intervention)
Jeong, 2005^64^	Republic of Korea	School/Educational	40 (2 arms)	Vulnerable group (Depression)	Adolescents (14–17)	Dance/Movement	Yes	Active	Dance Movement Therapy	DMT sessions designed around themes of body awareness, movement expressions, and integration of feelings	45-min sessions, 3 times a week, for 12 weeks	Passive control (Waitlist control)	Anxiety (SCL-90-R), Depression (SCL-90-R) (End of intervention)
Karakul, 2022^65^	Türkiye	Hospital/Clinical	33 (3 arms)	Vulnerable group (Cardiac catheterisation)	Older children (9–13)	Music	No	Receptive	Music Listening	Listening to “The Art of the Fuge by Johann Sebastian Bach” or self-selected music	Single session, started at sedation, continued during paediatric cardiac catheterisation procedure	Passive control (Usual care)	Anxiety (STAI), SBP, HR (End of intervention)
Kim, 2024^66^	Republic of Korea	School/Educational	50 (3 arms)	Vulnerable group (High-risk stress)	Adolescents (14–17)	Music	No	Active	Wind Instrument and Choral Training Program/Choral Training only	Combination of wind instrument performance (flute) and choral training with breathing exercises/Choral Training only	1 h 30 min per session, twice a week for 12 weeks	Passive control (Waitlist control)	QoL (WHOQOL), WB (PWI) (End of intervention)
Kirca, 2022^67^	Türkiye	School/Educational	100 (2 arms)	Vulnerable group (PMS)	Young adults (18–24)	Music	No	Receptive	Music Medicine	Listening to tailored music playlists based on their preferred genres or classical music	14 days, 3 times a week, 30 min each time	Passive control (No treatment)	QoL (WHOQOL) (End of intervention)
Koenig, 2013^121^	Germany	Hospital/Clinical	78 (2 arms)	Vulnerable group (Headache disorders)	Adolescents (14–17)	Music	Yes	Active	Specific Music Therapy	Participants engaged in a structured programme including music-assisted relaxation, imagery, improvisation, emotional expression, and role-play	6 sessions (90 min each) over 8 weeks +3 family sessions	Active control (Rhythm pedagogic programme)	Not included: Measured QOL (KIDSCREEN-27) but did not report summary measures
Lai, 2022^122^	USA	Community	58 (2 arms)	Vulnerable group (Cerebral palsy)	Adolescents (14–17)	Media (Music & Dance/Movement)	No	Active	Telehealth Movement-to-Music (M2M)	Adolescents with cerebral palsy participated in telehealth movement-to-music sessions	3 video sessions/week (48–100 min/week) for 4 weeks	Passive control (Waitlist)	Not included: QOL (neuroQOL) reported as change scores only
Lee, 2016^68^	Republic of Korea	School/Educational	64 (2 arms)	Healthy population	Young adults (18–24)	Music	No	Receptive	Music Listening	Listening to music for 20 min; music genres were primarily pop and gospel	Single session, 20 min	Passive control (No treatment)	SBP, HR (End of intervention)
Lindor, 2023^69^	Australia	Community	27 (2 arms)	Vulnerable group (ASD)	Older children (9–13)	Dance/Movement	No	Active	AllPlay Dance program	Community-based classical/contemporary dance program for autistic children, delivered by dance teachers in a group setting without explicit therapeutic goals	10-week program with eight 1-h dance sessions, one rehearsal, and a final performance	Passive control (Waitlist control)	QoL (PEDS-QoL) (End of intervention)
Malakoutikhah, 2020, 2023^123^^,^^124^	Islamic Republic of Iran	Education	46 (5 arms)	Healthy population	Young adults (18–24)	Music	No	Receptive	Music Listening (multiple genres)	Participants listened to different genres of music (Pop, Rock, Western Classical, Persian Traditional) or silence	15-min listening session per condition (crossover)	Passive control (Silence condition)	Not included: Measured Anxiety (STAI) but crossover study without correlation detail
Martin-Saavedra, 2020^70^	Colombia	School/Educational	52 (2 arms)	Vulnerable group (Primary dysmenorrhoea)	Young adults (18–24)	Music	No	Receptive	Music Listening	Listening to a C major musical composition with a tempo of 60 beats per minute, no percussion, and no lyric	29 min and 32 s, once during the first 12 h of menses	Passive control (Silence)	Anxiety (Zung), SBP, HR (End of intervention)
Mir, 2021^71^	Malaysia	School/Educational	30 (2 arms)	Vulnerable group (Pre-hypertension)	Young adults (18–24)	Music	No	Receptive	Music Listening	Listening to relaxing piano and flute music with eyes closed	30 min/day, 5 days/week for 4 weeks	Active control (Usual care)	SBP, HR (End of intervention)
Mittal, 2025^72^	India	Hospital/Clinical	210 (2 arms)	Vulnerable group (Dental treatment)	Younger children (5–8)	Literary arts	No	Receptive	Folk story	Listening to a folk story read to them in the waiting area	Single session before dental procedure	Active control (Dentistry modelling story)	Anxiety (MCDAS) (End of intervention)
Mohanasundari, 2023^73^	India	Hospital/Clinical	114 (3 arms)	Vulnerable group (Surgery)	Older children (9–13)	Music	No	Receptive	Music Medicine	Listening to preferred music through headphones	3 sessions, 15–30 min each, starting 12 h before anaesthesia induction	Passive control (Conventional treatment)	Anxiety (HAM-A) (End of intervention)
Mojahed, 2021^74^	Islamic Republic of Iran	Hospital/Clinical	48 (2 arms)	Vulnerable group (ADHD)	Older children (9–13)	Theatre/Performance	Yes	Active	Group Psychodrama	Psychodrama therapy tailored for children with ADHD to address psychological and social problems	2 h per week for 10 weeks	Passive control (Waitlist control)	Anxiety (SCAS) (End of intervention; ≥1-month follow-up)
Moula, 2020^75^	United Kingdom	School/Educational	62 (2 arms)	Vulnerable group (Emotional or behavioural difficulties)	Older children (9–13)	Multimodal	Yes	Active	Arts therapies	Arts therapy delivered in schools	1 h, once a week for 8 consecutive weeks	Passive control (Waitlist control)	QoL (HRQOL), WB (CORS) (End of intervention)
Muragod, 2024^76^	India	Hospital/Clinical	50 (2 arms)	Vulnerable group (Stress and anxiety)	Young adults (18–24)	Music	No	Receptive	Music Listening	Listening to Peter Sterling's album “The Angels Gift”	15 min, 4 times a week, for 3 weeks	Active control (Listening to a Guided Imagery audio by Johns Hopkins)	Anxiety (HAM-A) (End of intervention)
Nahir, 2024^77^	Türkiye	Hospital/Clinical	12 (3 arms)	Vulnerable group (Dental treatment)	Younger children (5–8)	Music	No	Receptive	Classical Music Listening	Listening to Classical instrumental music through a headset	Single session during dental treatment	Active control (Traditional behavioural guidance technique)	SBP, HR (End of intervention)
Nuvvula, 2015^78^	India	Hospital/Clinical	90 (3 arms)	Vulnerable group (Dental treatment)	Younger children (5–8)	Music	No	Receptive	Music distraction	Listening to preferred music using an MP3 player	Single session during local analgesia administration	Passive control (Usual care)	Anxiety (MCDAS), HR (End of intervention)
Osborn, 2023^79^	Kenya	School/Educational	235 (2 arms)	Vulnerable group (Depression/anxiety symptoms)	Adolescents (14–17)	Multimodal	No	Active	Pre-Texts	Arts-literacy intervention using texts to inspire art making followed by collective reflection	Daily sessions for a week, 1 h per session	Active control (Study skills intervention focused on notetaking, time management, and effective reading strategies.)	Anxiety (GAD-7), Depression (PHQ-8) (End of intervention; ≥1-month follow-up)
Ozturk, 2022^80^	Türkiye	School/Educational	170 (2 arms)	Healthy population	Young adults (18–24)	Visual Arts, Craft and Design	No	Active	Mindfulness-based mandala	Mindfulness-based mandala activity including breathing exercise, guided imagination and mandala drawing	Three 1-hr group sessions conducted over 3 weeks	Passive control (Waitlist control)	Anxiety (STAI), WB (SWBS) (End of intervention)
Pang, 2025^81^	Singapore	School/Educational	151 (3 arms)	Healthy population	Young adults (18–24)	Music	No	Receptive	Chinese Music Listening	Listening to Chinese instrumental music with/without dynamic theta to delta-frequency progression	Single session, 30 min	Active control (Listening to audiobook)	Anxiety (STAI) (End of intervention)
Piasai, 2018^82^	Thailand	Hospital/Clinical	40 (2 arms)	Vulnerable Group (Cancer)	Older children (9–13)	Multimodal	No	Active	Guided-Imagination and Drawing-Storytelling	Guided-imagination using classical music without lyrics for 30 min, followed by 30 min of drawing-storytelling	Single session, 60 min	Passive control (Usual care)	SBP, HR (End of intervention)
Porter, 2017^83^	United Kingdom	Community	251 (2 arms)	Vulnerable group (Emotional or behavioural difficulties)	Older children (9–13)	Music	Yes	Active	Music Therapy	Alvin model of ‘Free Improvisation' encouraging patients to create music and sound freely with support and encouragement	12 weekly sessions, each lasting 30 min	Passive control (Usual care)	Depression (CES-DC) (End of intervention)
Rafatjou, 2021^125^	Islamic Republic of Iran	Hospital/Clinical	132 (2 arms)	Vulnerable group (Dental treatment)	Younger children (5–8)	Music	No	Receptive	Music Listening	Children listened to music through headphone	Single session during dental visit	Active control (Alternative distraction)	Not included: Measured Anxiety (VPT), HR but do not present summary data by group
Rezazadeh, 2020^84^	Islamic Republic of Iran	Hospital/Clinical	60 (3 arms)	Vulnerable group (Burn diagnosis)	Older children (9–13)	Visual Arts, Craft and Design/Music	No	Active	Painting/Music	Painting with pencils and markers or listening to instrumental music, followed by conversations to express feelings	Daily session over 10 days, 45 min per session	Passive control (Usual care)	Anxiety (SCAS), Depression (CDI) (End of intervention)
Rodríguez-Jiménez, 2022^85^	Spain	School/Educational	37 (2 arms)	Healthy population	Young adults (18–24)	Dance/Movement	Yes	Active	Dance Movement Therapy	A body awareness program focussing on increasing body awareness, self-awareness, and well-being through structured sessions	10 sessions, 90 min each, twice a week	Passive control (No treatment)	WB (WHO-5) (End of intervention)
Sadeghi, 2023^126^	Islamic Republic of Iran	Hospital/Clinical	60 (2 arms)	Vulnerable group (Dental treatment)	Younger children (5–8)	Music & Literary arts	No	Receptive	Music/Story Listening	Children listened to music or an audio story	∼15–30 min per session during dental treatment (crossover)	Active control (Alternative distraction)	Not included: Procedural anxiety recorded but crossover study without correlation detail
Selçuk, 2022^86^	Türkiye	School/Educational	90 (3 arms)	Vulnerable group (Primary dysmenorrhoea)	Young adults (18–24)	Music	No	Receptive	Music Listening	Listening to a specifically composed song	Song duration: 29 min and 32, for 4 days (3 before menstruation and the first day of menstruation)	Passive control (No treatment)	Anxiety (STAI) (End of intervention)
Shahrbabaki, 2025^87^	Islamic Republic of Iran	Hospital/Clinical	64 (2 arms)	Vulnerable group (Surgery)	Older children (9–13)	Visual Arts, Craft and Design	No	Active	Mandala colouring	Colouring mandala patterns	Single session, 15–20 min before entering the operating room	Passive control (Usual care)	Anxiety (STAI) (End of intervention)
Sharda, 2018^88^	Canada	Hospital/Clinical	51 (2 arms)	Vulnerable group (ASD)	Older children (9–13)	Music	Yes	Active	Music Therapy	Use of improvisational approaches through song and rhythm to target social communication	8–12 weeks, 45-min individual weekly sessions	Active control (Behavioural intervention)	QoL (FQoL) (End of intervention)
Shawq, 2025^89^	Iraq	Hospital/Clinical	80 (2 arms)	Vulnerable group (Burns care)	Older children (9–13)	Music	No	Receptive	Music Listening	Listening to children's music, nature sounds, and classical music to alleviate pain and anxiety	10–15 min, daily during dressing changes	Passive control (Usual care)	Anxiety (STAI), HR (End of intervention)
Sheriff, 2023^127^	United Kingdom	Online	463 (2 arms)	Healthy population	Young adults (18–24)	Literary arts	No	Active	Cultural experience	Co-produced online intervention telling human stories behind art and artefacts	30 min a day for 3 days	Active control (Alternative intervention)	Not included: Anxiety (K10) measured but mean and SDs not reported
Siegel, 2016^128^	USA	Hospital/Clinical	25 (2 arms)	Vulnerable group (Hospitalisation)	Older children (9–13)	Visual Arts, Craft and Design	Yes	Active	Expressive Arts Therapy “Healing Sock Creatures”	Hospitalised children co-created healing sock creatures with expressive arts therapists	Single 90-min session during hospital stay	Passive control (Waitlist)	Not included: Cortisol measured but no summary data reported
Song, 2021^90^	Republic of Korea	School/Educational	99 (3 arms)	Healthy population	Young adults (18–24)	Music	No	Receptive	Healing Beats	Customised stress relief program using sound waves equivalent to the individual's resting heart rate and ECG waveform to induce relaxation	Single session, 40 min	Passive control (Placebo Group)	Anxiety (NRS), HR (End of intervention)
Soqia, 2025^91^	Syrian Arab Republic	School/Educational	69 (2 arms)	Healthy population	Young adults (18–24)	Music	No	Receptive	Jazz Music Listening	Listening to a standardised piece of Oriental Jazz music for 5 min via wired earphones	Single session, 5 min	Passive control (No treatment)	Anxiety (STAI) (End of intervention)
Stice, 2007^92^	USA	School/Educational	225 (6 arms)	Vulnerable group (Depression)	Young adults (18–24)	Literary arts	No	Active	Expressive writing	Writing about their emotional experiences	Three sessions, 45 min each	Passive control (Waitlist control)	Depression (BDI) (End of intervention; ≥1-month follow-up)
Stinley, 2015^129^	USA	Hospital/Clinical	40 (2 arms)	Vulnerable group (Needle procedure)	Older children (9–13)	Visual Arts, Craft and Design	No	Active	Mandala Creation	Children created mandalas on an iPad (drawing/colouring within a circular template)	Single session (∼5 min) before + during needle procedure	Passive control (Usual care)	Not included: HR measured but only change scores reported
Tan, 2021^93^	China	Hospital/Clinical	60 (2 arms)	Vulnerable group (Chronic disease)	Older children (9–13)	Visual Arts, Craft and Design	Yes	Active	Sandplay therapy	Making sandplay creations and explore inner world through Margaret Lowenfeld's sandplay therapy model	Six sessions, 1–2 times a week for 60–90 min each	Passive control (Usual care)	Anxiety (SAS), Depression (SDS) (End of intervention)
Nguyen, 2010^94^	Viet Nam	Hospital/Clinical	40 (2 arms)	Vulnerable group (Cancer)	Older children (9–13)	Music	No	Receptive	Music Medicine	Listening to self-selected music through earphones	Single session, started 10 min before and during a lumbar puncture procedure	Passive control (No treatment)	Anxiety (STAI), SBP, HR (End of intervention)
Ubrangala, 2022^95^	India	School/Educational	140 (4 arms)	Healthy population	Young adults (18–24)	Music	No	Receptive	Indian Music	Listening to pre-recorded Indian classical music modes played by flute/Bansuri with the drone (tanpura) in tonic in the background	Single session of 10 min	Active control (Natural sounds: birds chirping and flowing river)	Anxiety (STAI), SBP, HR (End of intervention)
Valizadeh, 2022^96^	Islamic Republic of Iran	Hospital/Clinical	102 (3 arms)	Vulnerable group (Hospitalised with fracture)	Older children (9–13)	Literary arts	No	Receptive	Story Listening/Story Listening and Reading	Listening (and reading) to stories via mp3 player and headphones	Single session, 30 min on the day of surgery	Passive control (No treatment)	Anxiety (RCMAS), HR (End of intervention)
Van der Heijden, 2019^131^	South Africa	Hospital/Clinical	197 (3 arms)	Vulnerable group (ER procedures)	Younger children (5–8)	Music	No	Receptive	Music Listening	Listening to recorded music of a repetitive and relaxing quality	Single session, during the procedure	Passive control (No treatment)	HR (End of intervention)
Wang, 2023^98^	China	Hospital/Clinical	181 (3 arms)	Vulnerable group (Surgery)	Younger children (5–8)	Music	No	Receptive	Music Listening	Listening to preferred music selected from a library using a multimedia audio system	Single session, 30–40 min	Passive control (Usual care)	Anxiety (mYPAS-SF), SBP, HR (End of intervention)
Xu, 2024^99^	China	School/Educational	40 (2 arms)	Vulnerable group (Depression/anxiety symptoms)	Young adults (18–24)	Dance/Movement	Yes	Active	Dance Movement Therapy	Structured program with twelve themes designed to address depression and anxiety	Three 6-h full day weekend sessions	Passive control (Usual care)	Anxiety (SAS), Depression (SDS) (End of intervention; ≥1-month follow-up)
Yan, 2019^100^	China	School/Educational	180 (3 arms)	Vulnerable group (Parental absence, at risk of bullying)	Older children (9–13)	Visual Arts, Craft and Design	No	Active	Painting activities	Used different theme painting activities to help students understand and cope with bullying	6 sessions over 3 months	Passive control (No treatment)	Anxiety (SCAS-R) (End of intervention)
Yi, 2024^130^	Nigeria	Education	450 (4 arms)	Vulnerable group (Kidnapping survivor with depression)	Adolescents (14–17)	Music & Visual Arts, Craft and Design	Yes	Active	Art and Music Therapy	Children received visual art therapy (e.g., drawing, painting, emotional expression) & music therapy (listening, singing, playing instruments)	12 hourly sessions (2 × weekly for 6 weeks)	Passive control (Usual care)	Not included: Depression (CDI) measured but no summary data reported
Yildirim, 2025^101^	Türkiye	Hospital/Clinical	90 (2 arms)	Vulnerable group (Hospitalisation)	Younger children (5–8)	Visual Arts, Craft and Design	No	Active	Toys making from medical materials	Creating toys using medical materials	Single session, for 30 min	Passive control (Usual care)	Anxiety (CSA) (End of intervention)
Yu, 2009^102^	China	Hospital/Clinical	60 (2 arms)	Vulnerable group (Cerebral Palsy, acupuncture)	Younger children (5–8)	Music	No	Receptive	Music Listening	Listening to their favourite music	Single session, for 30 min	Passive control (Listen to blank disc)	Anxiety (mYPAS), HR (End of intervention)
Yücel, 2024^103^	Türkiye	Hospital/Clinical	99 (3 arms)	Vulnerable group (Surgery)	Younger children (5–8)	Music	No	Receptive	Music Pillow	Listening to music they liked and chose themselves through a music pillow	Single session, 10 min before surgery	Passive control (Usual care)	Anxiety (CPMAS) (End of intervention)
Zamanifard, 2022^104^	Islamic Republic of Iran	Online/Virtual	40 (2 arms)	Vulnerable group (Type 1 Diabetes)	Older children (9–13)	Visual Arts, Craft and Design	No	Active	Virtual Directed Painting Therapy	Virtual painting therapy sessions where children express feelings through drawing	Six 2-h group sessions once a week for six weeks	Passive control (Usual care)	Anxiety (SCAS), Depression (SDS) (End of intervention)
Zhang, 2022^105^	China	School/Educational	71 (2 arms)	Vulnerable group (Depression)	Young adults (18–24)	Music	Yes	Active	Group Impromptu Music Therapy	Playing percussion instruments under music therapists' guidance to create personalised music	4 weeks, once a week	Passive control (Waitlist control)	Depression (BDI) (End of intervention)

ADHD, Attention-Deficit/Hyperactivity Disorder; ASD, Autism Spectrum Disorder; BAI, Beck Anxiety Inventory; BDI/BDI-II, Beck Depression Inventory/Beck Depression Inventory-II; CAM-S, Children's Anxiety Metre-State; CASS, Contextual Assessment of Social Skills; CBCS, Chota Bheem-Chutki Scale; CDI, Children's Depression Inventory; CES-DC, Centre for Epidemiologic Studies Depression Scale for Children; CFQ-R, Cystic Fibrosis Questionnaire-Revised; CORS, Child Outcome Rating Scale; CP, Cerebral Palsy; CP QOL-Child/OQL-Child, Cerebral Palsy Quality of Life Questionnaire for Children; CPMAS, Children's Perioperative Multidimensional Anxiety Scale; CSA, Children's State Anxiety scale; DASS-21/DASS21, Depression Anxiety Stress Scales-21; DMSRIA, Depressive Mood Self-Rating Inventory for Adolescents; DMT, Dance/Movement Therapy; ECG, electrocardiogram; FIS, Facial Image Scale; FQoL, Family Quality of Life scale; HADS/HADS-D, Hospital Anxiety and Depression Scale/Depression subscale; HAM-A, Hamilton Anxiety Rating Scale; HR, heart rate; HRQOL, Health-Related Quality of Life; ILQ, Inventory of Life Quality in Children and Adolescents; IV, intravenous; IWB, Index of Well-Being; KIDSCREEN-10/KIDSCREEN-27, KIDSCREEN health-related quality of life questionnaire (10-item/27-item); KINDL, KINDL Questionnaire for Health-Related Quality of Life in Children and Adolescents; LASA, Linear Analogue Scale Assessment; m-YPAS/YPAS/mYPAS-SF, (modified) Yale Preoperative Anxiety Scale (short form); MCDAS, Modified Child Dental Anxiety Scale; MHC-SF, Mental Health Continuum-Short Form; NRS, numeric rating scale; PedsQL/PEDS-QoL, Paediatric Quality of Life Inventory; PHQ-8, Patient Health Questionnaire-8; PMS, Premenstrual Syndrome; PMSS, Premenstrual Syndrome Scale; PWI, Personal Wellbeing Index; QoL/QOL, Quality of Life; RCMAS/RCMAS-2, Revised Children's Manifest Anxiety Scale (2nd Edition); RCT, randomised controlled trial; SAS, Self-rating Anxiety Scale; SBP, systolic blood pressure; SCARED, Screen for Child Anxiety Related Emotional Disorders; SCAS/SCAS-R, Spence Children's Anxiety Scale (Revised); SCL-90-R, Symptom Checklist-90-Revised; SD/SDs, standard deviation(s); SDS, Self-rating Depression Scale; ST-DEP, State-Trait Depression Questionnaire; STAI/STAIC/STAIC-T, State-Trait Anxiety Inventory/for Children/Trait subscale; SWBS/SWEMWBS, Short Warwick–Edinburgh Mental Well-being Scale; TMV, Therapeutic Music Video; VAS, Visual Analogue Scale; VPT, Venham's Picture Test; WB, Wellbeing; WHO-5, World Health Organisation Five Well-Being Index; WHOQOL/WHOQOL-BREF, World Health Organisation Quality of Life instrument (abbreviated version); Zung, Zung Self-Rating Anxiety/Depression Scale.

All six arts modalities were represented across the 100 included trials. Music was the most frequently investigated modality (n = 58), followed by visual arts, crafts, and design (n = 19); literary arts (n = 8); theatre and performance (n = 5); dance and movement (n = 5); and media-based interventions (n = 4). Seven interventions incorporated more than one modality. Nineteen studies evaluated arts therapies. Fifty-six interventions involved active engagement, whereas 44 involved receptive engagement. Intervention dosage ranged from single session to programmes comprising up to 52 weekly sessions.^35^ Session durations ranged from approximately 2 min to 6 h, with over half lasting between 30 and 90 min. Among multi-session interventions, programmes were typically delivered over approximately 6 weeks (IQR 3–10 weeks) and comprising a median of 8 sessions (IQR 6–10 sessions). Seventy-eight studies used passive control comparators, including usual care, no treatment, or waitlist, and 22 studies used active comparators such as alternative interventions.

Across included studies, mental health outcomes (depression, anxiety, wellbeing, and quality of life) were assessed predominantly using self-report scales (e.g., Beck Depression Inventory (BDI), Generalised Anxiety Disorder-7 (GAD-7)); a smaller number used observer-rated behavioural scales, particularly in younger children undergoing medical or dental procedures (e.g., Venham's Picture Test); and two studies used a clinician-administered scale (Hamilton Anxiety Rating Scale (HAM-A)). None of the included studies used a clinical diagnostic interview as the outcome measure.

The meta-analysis included 77 RCTs that reported sufficient detail to enable meta-analysis and acknowledge the risk of bias from selective outcome reporting. For example, some excluded studies reported only the ‘statistically significant’ outcomes in detail, suppressing the inclusion of non-significant results.

Four studies were judged to be at low risk of bias, while the remainder were rated as having some concerns or high risk, primarily because most studies lacked a detailed pre-specified protocol or analysis plan and did not report on allocation concealment. Although all included studies employed validated outcome measures, in most cases participants and carers could not be blinded due to the nature of the intervention. Consequently, self-reporting of depression, anxiety, wellbeing, and QoL led us to rate the domain relating to the measurement of these outcomes as having at least ‘some concerns’ (Fig. 2; see also Supplementary Figures S16 and S17 for traffic light plot of risk-of-bias assessments of mental health and biomarker outcomes, respectively).

**Fig. 2:**
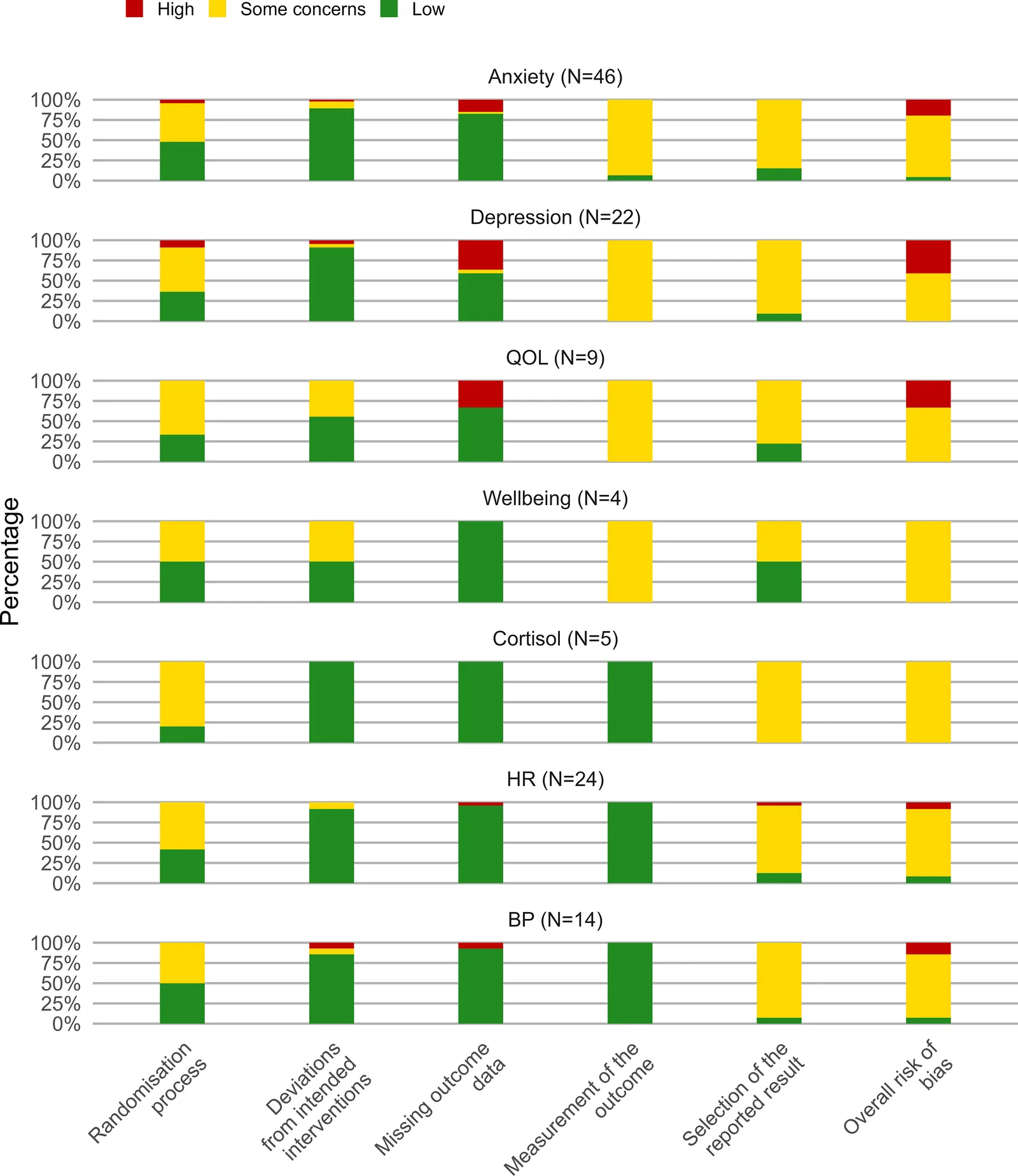
**Risk of bias assessment.** Note: QOL, Quality of Life; HR, heart rate; BP, blood pressure.

As shown in Fig. 3, there were moderate to very large effects on the primary outcomes at the end of the intervention (T1). Evidence from 23 comparisons from 22 trials relating to 1655 participants suggested that arts-based interventions reduced depression scores at T1 (median 8 weeks, IQR 4–10 weeks) with a very large effect (on average 1.06 SMD, 95% CI 0.45–1.67, *I*^2^ = 97%).However, heterogeneity was also considerable indicating inconsistency between study results (Supplementary Figure S1).

**Fig. 3:**
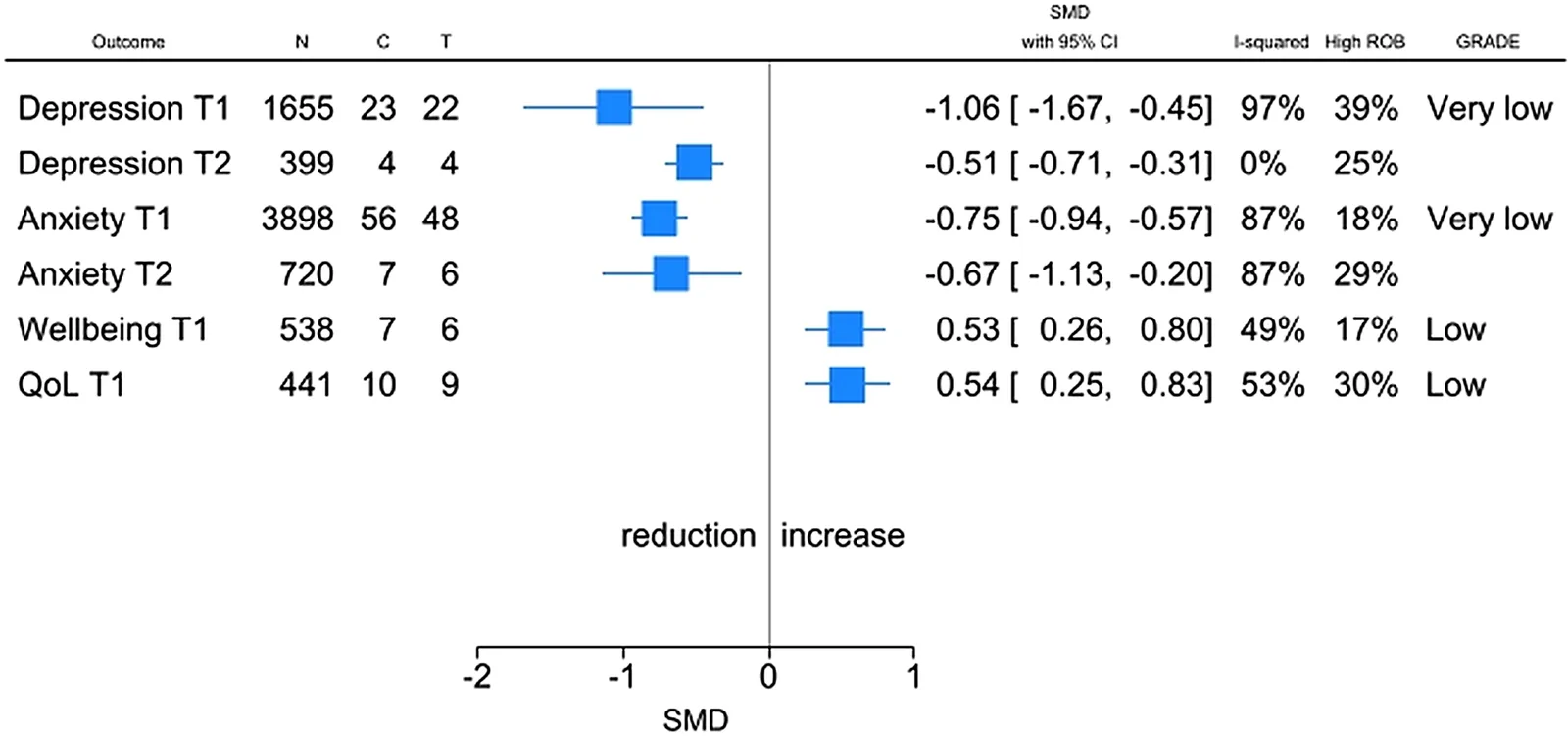
**Meta-analysis results for the effects of arts-based interventions on mental health outcomes (SMDs).** Note: N, participants; C, comparisons; T, trials; SMDs, Standardised Mean Differences; T1, immediately post-intervention; T2, follow-up at least one month post-intervention; QoL, Quality of Life; ROB, Risk of Bias; GRADE, Grading of Recommendations Assessment, Development and Evaluation. Horizontal lines represent 95% confidence intervals. A reduction in SMD reflects lower depression or anxiety scores, and an increase reflects higher wellbeing or QoL scores; in both cases this favours the arts-based intervention over the comparator. See Supplementary Figures S1–S4 for forest plots with outcome-specific ‘favours’ labelling.

Evidence from 56 comparisons from 48 trials relating to 3898 participants suggests that arts-based interventions reduced anxiety scores at T1 (median 6 weeks, IQR 2–10 weeks) with a large effect (on average 0.75 SMDs, 95% CI 0.57–0.94; *I*^2^ = 87%); high heterogeneity was also present (Supplementary Figure S2). Moderate effects were found for wellbeing and QoL, but the evidence is derived from fewer comparisons.

Evidence from seven comparisons from six trials of 538 participants suggests that arts-based interventions had a moderate effect on wellbeing scores (0.53 SMD; 95% CI 0.26–0.80, *I*^*2*^ = 49%) at T1 (median 6.5 weeks, IQR 4–8 weeks) and based on evidence from ten comparisons relating to nine trials and 441 participants, arts-based interventions appeared to have a moderate effect on QoL (0.54 SMDs; 95% CI 0.25–0.83, *I*^*2*^ = 53%) at T1 (median 10 weeks, IQR 8–12 weeks). Moderate heterogeneity was present for both of these two measures (Supplementary Figure S3 and S4).

There were sustained changes in depression and anxiety at follow-up (T2, at least one month after the end of the arts-based intervention). Evidence from four trials encompassing 399 participants suggested that arts-based interventions reduced depression scores at T2 (median 2 months, IQR 1–4 months) with a moderate effect (on average 0.51 SMD, 95% CI 0.31–0.71, *I*^*2*^ = 0%), and based on seven comparisons from six trials it was suggested that arts-based interventions reduced anxiety at T2 (median 2 months, IQR 1–4 months) with a moderate to large effect (0.67 SMDs, 95% CI 0.20–1.13, *I*^*2*^ = 87%), While there was very low heterogeneity for depression, high heterogeneity was present for anxiety, suggesting inconsistency between results. No T2 data was available for other outcomes (see Supplementary Figure S1–S7 for further details).

Subgroup analysis revealed effect sizes for depression were larger for passive control than active control (SMD = 1.15 vs SMD = 0.22, *p* = 0.01), and for young adults (18–24) more than other age groups, however the result relating to age was sensitive to the inclusion of 3 studies with very large effect sizes (see Supplementary Figures S8a and b). Despite the heterogeneity, there was no clear indication that effect size varied across modalities or between arts therapy and other arts-based interventions (Supplementary Figure S8c). Similarly, t effect sizes for depression did not appear to differ between vulnerable and healthy populations, engagement type, number of sessions or risk of bias.

Effect sizes for anxiety varied by age groups, arts modality, control type, and dosage (Supplementary Figures S9a-c): larger effect sizes were observed for anxiety among children (5–8 and 9–13) and young adults (18–24) compared to adolescents (14–17) (SMD = 0.84, SMD = 0.87, SMD = 0.63 vs SMD = 0.25); for theatre/performance (SMD = 1.33) and multimodal interventions (SMD = 1.26) compared to music (SMD = 0.73), visual arts (SMD = 0.75), dance/movement (SMD = 0.49) or literary arts (SMD = 0.13); and when there were passive controls compared to active controls (SMD = 0.87 vs SMD = 0.35). Effect sizes for interventions delivered over 13 or more sessions tended to be smaller than those delivered over a shorter duration, but this result was sensitive to the inclusion of one study with a very large effect size. There was no clear indication that effect sizes varied between arts therapy and other arts-based interventions, vulnerable and healthy population, type of engagement, or risk of bias.

There were no significant subgroup differences for wellbeing outcomes (see Supplementary Figures S10). For QoL, those aged 9–13 showed larger effects than other age groups (SMD = 0.81 vs SMD = 0.03, SMD = 0.22) and studies with active controls also showed a larger effect than studies with passive controls (SMD = 1.25 vs SMD = 0.44). No other subgroup differences were detected (see Supplementary Figures S11).

Fig. 4 provides an overview of the biometric data reviewed in this study. Data from four comparisons across four studies involving 261 participants examining cortisol changes were inconclusive (SMD −0.24; 95% CI −0.50 to 0.01; *I*^*2*^ = 8%). Data from 15 comparisons from twelve trials, relating to 693 participants, suggests that arts intentions reduced systolic blood pressure by an average of 4.04 mmHg (95% CI 0.74–7.3; *I*^*2*^ = 71%). Data from 29 comparisons from 25 studies based on 1667 participants suggests that arts-based interventions reduced heart rate by an average of 4.53 beats per minute (95% CI 2.31–6.76; *I*^*2*^ = 77%) (see Supplementary Figure S5–S7).

**Fig. 4:**
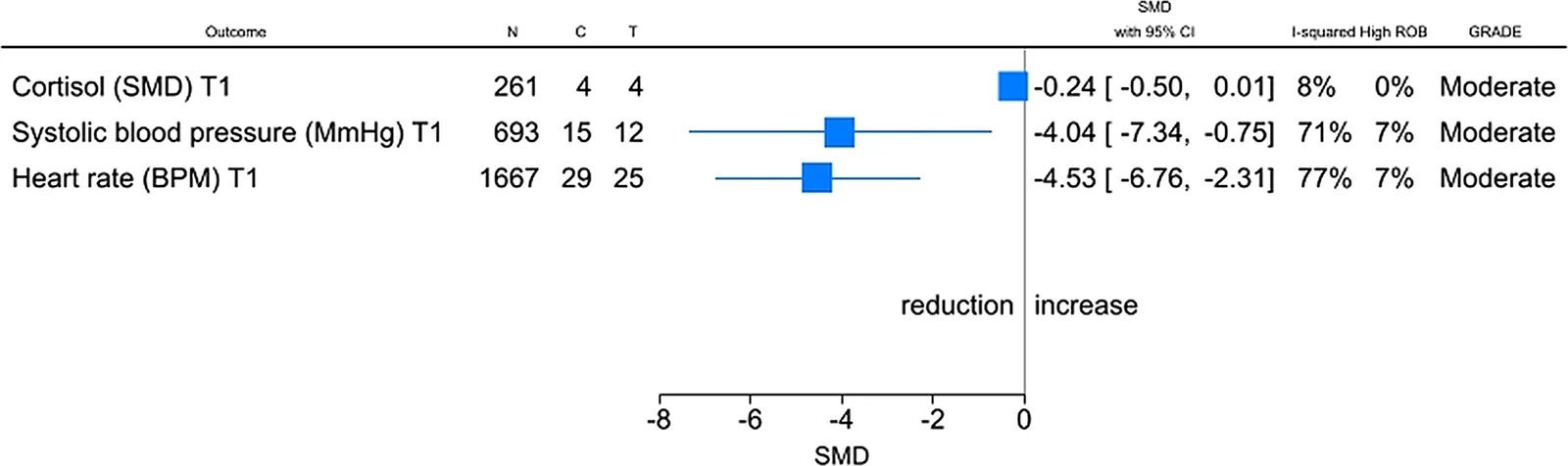
**Meta-analysis results for the effects of arts-based interventions on biomarkers.** Note: N, participants; C, comparisons; T, trials; SMD, Standardised Mean Difference; T1, immediately post-intervention; MmHg, millimetres of mercury; BPM, beats per minute; ROB, Risk of Bias; GRADE, Grading of Recommendations Assessment, Development and Evaluation. Horizontal lines represent 95% confidence intervals. See also Supplementary Figures S5–S7 for forest plots on biomarkers.

Subgroup analyses suggested the effect size for systolic blood pressure was largest for those aged 9–13 years old (see Supplementary Figures S13). Contrarily, they were the smallest in studies for 18–24-year-olds when compared with other age groups. Effect sizes tended to be larger in studies using interventions with active engagement than those where engagement was receptive and in multimodal interventions compared to music.

Heart rate varied by type of arts modality (literary interventions tended to reduce heart rate the least), population (in studies with vulnerable groups had higher reduction of heart rate than with healthy populations) and age (there was a larger effect size with children than with adolescents and young adults). Studies rated as low risk of bias tended to have smaller effect sizes than those rated as ‘some concerns’ or ‘high’ (see Supplementary Figures S14).

There was no evidence that effect sizes for cortisol varied by subgroup (see Supplementary Figures S12).

Two sets of post-hoc sensitivity analyses were performed. First, removing studies with extremely high effect sizes (SMD greater than three) attenuated the effect size for depression from 1.06 (95% CI 0.45–1.67, *I*^2^ = 97%) to 0.58 (95% CI 0.32–0.85, *I*^2^ = 83%), and for anxiety from 0.75 (95% CI 0.57–0.94, *I*^2^ = 87%) to 0.71 (95% CI 0.54–0.88, *I*^2^ = 84%); both results remained statistically significant with a moderate-to-large effect size. Second, removing studies judged to be at high risk-of-bias produced comparable estimates for depression (1.06–0.70, 95% CI 0.21–1.20) and anxiety (0.75–0.71, 95% CI 0.52–0.90). Sensitivity analyses made only small changes to estimates for quality of life and the biomarker outcomes; the wellbeing estimate was less robust, with its confidence interval crossing the null following removal of a high-risk-of-bias study (see Supplementary Figures S8b and 9b and Supplementary Table S3 for details).

Funnel plots (Supplementary Figure S15) revealed possible evidence of publication bias or selective outcome reporting for the outcomes of depression, anxiety and heart rate; there was asymmetry due to several extreme positive results while extreme negative results of a similar magnitude were missing. Note that a funnel plot for cortisol was not produced due to there only being four studies in the analysis of this outcome.

Our GRADE rating of the certainty of results for depression and anxiety was downgraded to very low certainty due to serious inconsistency indicated by the very high *I*-squared values, imprecision (wide confidence intervals) and risk of bias concerns. Wellbeing and quality of life were downgraded to low due to imprecision and concerns about risk of bias. Moderate certainty was demonstrated in the assessment of the results relating to heart rate and blood pressure. See Supplementary Material for further details.

## Discussion

This review demonstrates that arts-based interventions are associated with substantial improvements in MH outcomes for CYP. Large effect sizes were observed for reductions in both depression and anxiety, with the limited follow-up data available indicating only a modest attenuation of these effects one month after the intervention. However, due to the substantial heterogeneity and imprecision, particularly for the outcome of depression, these effect sizes should not be interpreted as representative of arts-based interventions in general; rather, they should be read as evidence that, on average, the interventions included in the trials in this review were beneficial. Moderate improvements in wellbeing and QoL were also identified, although no studies reported longer-term follow-up for these outcomes. Subgroup analyses in each of the main outcomes suggest that effect sizes varied with regards to age (depression, anxiety, and QoL), arts modality (anxiety), passive or active controls (depression, anxiety, and QoL).

Findings from biomarkers provide emerging but still preliminary evidence regarding the potential regulatory effects of arts-based interventions on CYP. While reduction in cortisol levels was small and its clinical relevance remains uncertain, consistent outcomes were observed in systolic blood pressure and resting heart rate. Notably, larger reductions in blood pressure were reported in interventions involving active compared with receptive engagement. Although the available data for CYP were limited to short-term effects, reductions in systolic blood pressure and resting heart rate at the population level are typically associated with lower long-term cardiovascular risk^132^^,^^133^ and improved fitness.^134^ These patterns suggest that arts participation may contribute to broader physiological regulation alongside psychological benefits.

The few studies that do report follow-up data suggest that benefits may be sustained. For example, the Arts for the Blues school-based studies reported positive effects up to six months after the end of the intervention.^75^ These results however, should be cautiously interpreted given the limited longer-term evidence available.

Overall, findings in this review of arts-based interventions provide comparable evidence to talk therapy intervention (CBT in particular) on MH outcomes amongst school-aged CYP.^5^^,^^18^^,^^19^^,^^135^ The effects reported for depression and anxiety exceed those reported for talking therapies and CBT in similar age group,^5^^,^^135^ and are comparable to the high effects reported in recent arts therapies research.^18^^,^^19^ These findings align with results from our earlier umbrella review,^16^ which reported large effects for arts-based interventions for a wider age group with MH conditions^16^ that surpassed those reported in CBT umbrella reviews.^136^ These findings, however, lack head-to-head comparisons, which will be an important direction for future research exploring optimal care strategies for CYP MH.

Across outcomes, larger effects were observed in the 9–13 age group, vulnerable populations, multimodal interventions, and active participation, with no differences detected between arts therapies and non-arts therapies. The 9–13 age group showed particularly strong gains in anxiety, QoL, and blood pressure, aligning findings from other meta-analyses that arts-based interventions reduce internalising symptoms and physiological arousal in children and adolescents.^18^^,^^137^ This suggests that middle childhood may be an especially responsive developmental period for arts-based intervention. Larger improvements in heart rate observed in vulnerable groups compared with healthy populations suggest a greater capacity for change when baseline dysregulation is elevated, highlighting the value of positioning arts-based interventions as targeted support for those with higher psychosocial or physiological need.

Multimodal approaches outperformed most single-modality formats in anxiety outcomes, suggesting that simultaneously engaging sensory, social, and movement-based processes provides a richer and more effective therapeutic pathway than any single modality alone.^138^^,^^139^ This finding has direct implication for practice and needs to inform associated training of practitioners across artforms; whilst specific skills in an art form are important for excelling as an artist, MH appears to improve more substantially when diverse experiences are offered.

Although our subgroup analyses did not identify significant differences between arts therapies and non-arts therapies, psychological literature suggest that targeted, clinician-delivered interventions report greater improvements than those delivered by non-MH specialists.^5^ The absence of this subgroup difference is constrained by a substantial imbalance in the evidence base (64 of 77 trials involved arts-based interventions not classified as arts therapies, including 28 perioperative dominated by receptive music-listening interventions), highlighting the under-representation of arts therapies or other healthcare professionals who use arts in their practice. This signals the need for further research with adequately powered comparisons and clearer distinctions between arts therapies and non-arts therapies interventions.

This review is subject to several limitations that should be considered when interpreting the findings. A key limitation is that the majority of studies presented with ‘some concerns’ about risk of bias, predominantly due to the use of self-reported outcomes, lack of blinding, absence of a detailed pre-specified protocol or analysis plan, and lack of reporting on allocation concealment, limiting high confidence in the results. Given the nature of arts-based intervention, blinding of participants was not possible, a limitation also common in psychological trials.^140^ To address this, we examined biomarkers as lower-risk-of-bias measures, which generally corroborated the self-reported mental health outcomes. Still, publication bias or selective outcome reporting was suggested by funnel plot asymmetry for depression, anxiety and heart rate. These challenges are compounded by the absence of an appraisal tool designed for arts-based RCTs, which often depend on self-report and participants' awareness of their group allocation. Consequently, some biases are inherently difficult to minimise in this field.

Another key limitation is the substantial heterogeneity in the results, arising from several sources. Diverse arts modalities and intervention approaches (clinical heterogeneity) and multiple outcome measures for depression, anxiety, wellbeing and QoL (statistical heterogeneity) were a major contributor. Comparator conditions also varied, with 78 studies using passive controls (usual care, no treatment, or waitlist) and 22 using active comparators. Our ‘vulnerable’ population category combined a wide range of conditions (see Methods for full definition), including studies of dental anxiety, which are typically brief, single-session, procedure-related distraction interventions pooled within the same subgroup as longer, structured interventions for diagnosed mental health conditions. Variable duration of interventions (T1) and post-intervention follow-up times (T2) across studies were also unavoidable, and this variability might have affected the results. Finally, the diversity of art forms grouped within each modality category, and within the broader arts-based intervention category as a whole, means a single pooled or subgroup effect size may not adequately represent any specific art form or therapeutic approach.

Although our subgroup analyses explained some of this heterogeneity, substantial heterogeneity remained within subgroups, and pooling together such diverse interventions limited our ability to fully explain it, as a result limiting the conclusions that can be drawn. Removing the three studies with extremely high effect sizes (SMD greater than 3) and studies judged to be at high risk-of-bias considerably attenuated the effect size for depression from 1.06 to 0.58 and 0.70 respectively, although this result remained statistically significant with a moderate-to-large effect size. Further high-quality longitudinal research utilising agreed intervention lengths and post-intervention follow-up times is needed to clarify immediate effects and their durability, as such agreements amongst research scholars in this field are not yet available.

The review is also limited by our decision to restrict inclusion to RCTs, which has limited the breadth of the evidence captured, as research in arts-based interventions taking place in the community, schools or other non-clinical settings does not typically employ RCT designs. Adopting assumptions that are often different to the medical model and operating with precarious funding are some reasons for the limited presence of RCTs in this area.

This study provides a clear evidence-base for the contribution of arts-based interventions to supporting the MH of CYP. To our knowledge, this study is one of the most comprehensive meta-analyses of RCTs examining arts-based interventions for CYP, incorporating both psychological outcomes and relevant physiological biomarkers. By integrating biomarker data, which are typically at lower risk-of-bias, we were able to partially validate self-reported outcomes and strengthen confidence in the overall pattern of findings. The inclusion of diverse modalities and multiple comparison groups allowed for detailed subgroup analyses, and sensitivity analysis enhancing the robustness of the conclusions. These methodological features offer an important map of the field that has not been available as yet.

The significant increase of MH rates amongst CYP, and the general preference of arts-based interventions by young people themselves, potentially reaching underserved communities, bring further significance in the findings from this review. However, it remains important for policymakers and clinicians to distinguish between arts with therapeutic intent and arts therapies delivered by appropriately trained professionals involving processes that are often more complex than engagement in arts activities alone when considering service design, resource allocation, workforce planning and training. The role of artists is increasingly relevant to current policy discussions, including the potential role of community-based arts programmes (e.g., in youth community hubs) in social prescribing that can reduce pressure on clinical services. Qualified healthcare and/or MH practitioners (arts therapists, creative counsellors, OTs or nurses) should also be considered not only in order to enrich service provision and diversify the workforce but also, crucially, to meet diverse CYP needs safely.

However, further investment in research is needed to increase the quality of current evidence, including studies that examine delivery features, mechanisms, and contextual factors across both clinical, educational and community settings to provide a more comprehensive understanding of what drives therapeutic benefit in specific arts interventions. This will lead to a research-informed use of the arts and arts therapies as MH support for CYP supporting prevention, early intervention, treatment and management decisions, and thus, offering a promising additional solution to a MH problem that needs considerable and fresh attention.

## Contributors

PSC led the data curation and project administration and contributed to the conceptualisation, investigation, methodology, validation, visualisation, writing (original draft preparation and review & editing), and provided supporting input to the formal analysis. SR contributed to the conceptualisation, data curation, investigation, methodology, software, validation, visualisation, and writing (original draft preparation and review & editing) of the study. EB contributed to the conceptualisation, data curation, investigation, methodology, software, and validation, and provided supporting contributions to writing (original draft preparation and review & editing). WL provided supporting contributions to validation, visualisation, and writing (original draft preparation and review & editing).

NS contributed to conceptualisation, resources, software, supervision, validation, and writing (review & editing). NF contributed to manuscript review. VK, who served as the principal investigator and guarantor, led the conceptualisation, funding acquisition, investigation, resources, software, supervision, and original draft writing, and contributed to data curation, methodology, project administration, validation, and manuscript review and editing.

PSC and SR accessed and verified the underlying data.

All authors read and approved the final version of the manuscript.

The guarantor accepts full responsibility for the work and the decision to publish. The corresponding author attests that all listed authors meet authorship criteria and that no others meeting the criteria have been omitted.

## Data sharing statement

No additional data are available. This study is a systematic review and meta-analysis based on previously published data, all of which are publicly accessible through the original sources cited in the manuscript.

## Declaration of interests

All authors have completed the ICMJE uniform disclosure form at http://www.icmje.org/disclosure-of-interest/and declare: PSC and VK are supported by research funding from United Kingdom Research and Innovation and the Arts and Humanities Research Council for the Arts4Us project granted to the Research Centre for Arts and Wellbeing at Edge Hill University, SR and WL are supported by University of Manchester with funding from the NIHR. Specifically, WL's institution has received funding from the NIHR Pre Doctoral Fellowship to cover his salary, while SR's institution has received funding from the NIHR from multiple grants to cover part of her salary. SR is also in NIHR trial steering committee and a member of the EU-PAL data monitoring committee and a member of the NIHR RfPB funding panel. She has also received a £500 honorarium from the Journal of Maternal and Child Nutrition for statistical editing. EB and NS are supported by Jameel Arts & Health lab for the submitted work, NF is supported by a grant from the Community Jameel for the submitted work; no financial relationships with any organisations that might have an interest in the submitted work in the previous three years; no other relationships or activities that could appear to have influenced the submitted work.

## Footnotes

e Disclaimer: The authors alone are responsible for the views expressed in this article and they do not necessarily represent the views, decisions or policies of the institutions with which they are affiliated.
