# Supplementary material for: The effects of the arts and arts therapies on mental health outcomes of children and young people: a systematic review and meta-analysis
Source: eClinicalMedicine. 2026 Oct 15;101:104261. doi: 10.1016/j.eclinm.2026.104261 (PMC13694774; doi:10.1016/j.eclinm.2026.104261)
Supplement: Supplementary file 1 [file mmc1.docx]

**Title: The effects of the arts and arts therapies on mental health outcomes of children and young people: A systematic review and meta-analysis**

**Supplementary Materials**

Table of Contents

FOREST PLOTS FOR MENTAL HEALTH OUTCOMES	3

Supplementary Figure 1. Forest plot for depression.	3

Supplementary Figure 2. Forest plot for anxiety.	4

Supplementary Figure 3. Forest plot for Wellbeing.	5

Supplementary Figure 4. Forest plot for quality of life.	5

FOREST PLOT FOR BIOMARKERS	6

Supplementary Figure 5. Forest plot for cortisol.	6

Supplementary Figure 6. Forest plot for systolic blood pressure.	6

Supplementary Figure 7. Forest plot for heart rate.	7

SUBGROUP ANALYSES FOR MENTAL HEALTH OUTCOMES	8

Supplementary Figure 8a. Subgroup analysis for depression.	8

Supplementary Figure 8b. Subgroup analysis for depression with 3 studies with high effect sizes removed (Citil, Gee, Zhang)	9

Supplementary Figure 8c. Frequency of intervention modality by arts therapy status, depression meta-analysis (end of intervention)	10

Supplementary Figure 9a. Subgroup analysis for anxiety.	11

Supplementary Figure 9b. Subgroup analysis for anxiety with one study with very high effect size removed (Citil)	12

Supplementary Figure 9c. Frequency of intervention modality by arts therapy status, anxiety meta-analysis (end of intervention)	13

Supplementary Figure 10. Subgroup analysis for wellbeing.	14

Supplementary Figure 11. Subgroup analysis for quality of life	15

Supplementary Figure 12. Subgroup analysis for cortisol.	16

Supplementary Figure 13. Subgroup analysis for systolic blood pressure	17

Supplementary Figure 14. Subgroup analysis for resting heart rate.	18

FUNNEL PLOTS	19

Supplementary Figure 15. Funnel plots	19

RISK OF BIAS	20

Supplementary Figure 16. Risk assessment of mental health outcomes (anxiety, depression, quality of life)	20

Supplementary Figure 17. Risk of Bias assessment for biomarker outcomes (heart rate, blood pressure, cortisol)	23

SUPPLEMENTARY TABLES	24

Supplementary Table 1. A sample search strategy.	24

Supplementary Table 2. Definitions of Study Subgroups	25

Supplementary Table 3. Removing studies judged high risk of bias.	26

Supplementary Table 4. Summary of overall findings with GRADE Assessment	27

Supplementary Table 5. Data extraction columns and coding framework used in Elicit	29

Supplementary Table 6. List of included studies	30

Supplementary Table 7. List of excluded studies with reasons	34

Supplementary Table 8. List of eligible studies from top-up search on 30/4/2026 (not included in this review)	47

# FOREST PLOTS FOR MENTAL HEALTH OUTCOMES

## Supplementary Figure 1. Forest plot for depression.

## Supplementary Figure 2. Forest plot for anxiety.

## Supplementary Figure 3. Forest plot for Wellbeing.

## Supplementary Figure 4. Forest plot for quality of life.

# FOREST PLOT FOR BIOMARKERS

## Supplementary Figure 5. Forest plot for cortisol.

## Supplementary Figure 6. Forest plot for systolic blood pressure.

## Supplementary Figure 7. Forest plot for heart rate.

# SUBGROUP ANALYSES FOR MENTAL HEALTH OUTCOMES

## Supplementary Figure 8a. Subgroup analysis for depression.

## Supplementary Figure 8b. Subgroup analysis for depression with 3 studies with high effect sizes removed (Citil, Gee, Zhang)

## Supplementary Figure 8c. Frequency of intervention modality by arts therapy status, depression meta-analysis (end of intervention)  (n = 23 comparisons)

Striped area = studies coded as arts therapy; solid area = studies coded as not arts therapy

Proportion of arts therapy within each modality:

- Music - 4 out of 12
- Visual arts, craft and design – 1 out of 5
- Literary arts – 0 out of 1
- Multimodal – 0 out of 3
- Dance/movement – 2 out of 2

## Supplementary Figure 9a. Subgroup analysis for anxiety.

## Supplementary Figure 9b. Subgroup analysis for anxiety with one study with very high effect size removed (Citil)

## Supplementary Figure 9c. Frequency of intervention modality by arts therapy status, anxiety meta-analysis (end of intervention) (n = 56 comparisons)

Striped area = studies coded as arts therapy; solid area = studies coded as not arts therapy

Proportion of arts therapy within each modality:

■ Music - 2 out of 30

■ Visual arts, craft and design – 1 out of 11

■ Literary arts – 2 out of 5

■ Theatre/performance – 1 out of 5 studies

■ Multimodal – 0 out of 3

■ Dance/movement – 2 out of 2

## Supplementary Figure 10. Subgroup analysis for wellbeing.

*Note: proportion of arts therapy within each modality: music – 1 out of 4; dance/movement – 1 out of 1; multimodal – 1 out of 1; visual arts, craft and design – 0 out of 1.*

## Supplementary Figure 11. Subgroup analysis for quality of life

*Note: proportion of arts therapy within each modality: music – 2 out of 6; multimodal – 1 out of 2; dance/movement – 0 out of 1; visual arts, craft and design – 0 out of 1.*

**SUBGROUP ANALYSES FOR BIOMARKERS**

## Supplementary Figure 12. Subgroup analysis for cortisol.

*Note: None of the studies contributing to these analyses were coded as arts therapy.*

## Supplementary Figure 13. Subgroup analysis for systolic blood pressure

*Note: None of the studies contributing to these analyses were coded as arts therapy.*

## Supplementary Figure 14. Subgroup analysis for resting heart rate.

*Note: None of the studies contributing to these analyses were coded as arts therapy.*

# FUNNEL PLOTS

## Supplementary Figure 15. Funnel plots

# RISK OF BIAS

## Supplementary Figure 16. Risk assessment of mental heatlh outcomes (anxiety, depression, wellbeing, quality of life)

## Supplementary Figure 17. Risk of Bias assessment for biomarker outcomes (heart rate, blood pressure, cortisol)

# SUPPLEMENTARY TABLES

## Supplementary Table 1. A sample search strategy.

| Database: EMBASE, limiter: 1/1/2005 to present | |
| --- | --- |
| **#1** | (art OR arts OR artistic OR paint* OR drawing OR pottery OR ceramic OR kintsugi OR sculpt* OR craft* OR collage OR mosaic OR illustration OR printmaking OR jewellery OR jewelery OR metalworking OR textiles OR fashion OR music* OR singing OR choral OR choir OR drumming OR song* OR “song writing” OR “music composition” OR “lyric analysis” OR “sound therapy” OR vibroacoustic OR concert OR orchestra OR “musical instruments” OR “movement therapy” OR “authentic movement” OR tango OR flamenco OR “five rhythms” OR “5 rhythms” OR 5rhythms OR biodanza OR somatic* OR butoh OR capoeira OR ballet OR bellydance OR “belly dance” OR salsa OR drama OR dramatherapy OR psychodrama OR theat* OR puppetry OR clowning OR actor* OR actress* OR “role play” OR improv OR improvisation OR “mask making” OR opera OR circus OR comedy OR poetry OR poems OR bibliotherapy OR “creative writing” OR “spoken word*” OR zines OR storytelling OR fairytale* OR screenwriting OR “screen writing” OR “fairy tale*” OR journaling OR filming OR “film making” OR cinema OR animation OR “video making” OR “computer graphics” OR “play therapy” OR “mixed medium” OR “mixed media” OR creativ* OR festival OR museum OR gallery OR exhibition OR carnival):ti,ab,kw |
| **#2** | (Child OR Children OR Kid OR Kids OR Young OR Youth OR Adolescen* OR Teen* OR Pupil* OR Student* OR Boy* OR Girl* OR Juvenile OR "Young adult*" OR "Emerging adult*" OR "Pre adult*" OR Preteen OR pre-teen OR Schoolchild OR Learner OR Undergraduate OR pre-adult OR “Gen Z” OR “Gen alpha” OR "Early adulthood" OR Prepubescen* OR Pubescen*):ti,ab,kw |
| **#3** | #1 AND #2 |
| **#4** | ("antiretroviral therapy" OR "atraumatic restorative treatment" OR antiretroviral OR anti-retroviral OR "assisted reproductive treat*" OR (assisted NEAR/1 reproduct* NEAR/1 technolog*) OR "accelerated resolution therapy" OR "anti-retro viral therap*" OR (Atraumatic NEAR/1 Restorat* NEAR/1 Treatment*) OR (accelerated NEAR/1 resolution NEAR/1 therapy) OR “constraint-induced movement therapy”  OR "State of the art" OR "martial art*" OR "State of art" OR "the art of"):ti,ab,kw OR ("systematic review" OR “meta-analysis” OR “meta analysis”):ti |
| **#5** | #3 NOT #4 |
| **#6** | ("randomi* control* trial*" OR "clinical trial*" OR “randomi* control* stud*” OR “randomi* trial*” OR “cluster* trial*” OR stepped-wedge OR “stepped wedge” OR RCT):ti, |
| **#7** | #5 AND #6 |

## Supplementary Table 2. Definitions of Study Subgroups

| **Subgroup Variable** | **Variation** |
| --- | --- |
| Population needs | Population *needs* were categorised into the presence or absence of a condition, thus grouped as either vulnerable or healthy populations respectively.   - Vulnerable group: CYP with or at risk of depression, anxiety, and other MH problems; physical or neurodevelopmental conditions (ASD, ADHD, cerebral palsy); physical illness (chronic pain, cancer, diabetes); hospitalised or undergoing medical/dental procedures. - Healthy populations: Participants with no apparent needs. |
| Age groups | *Age* groups were defined according to the age range, mean age, and midpoint and classified as:   - Younger children (5-8) - Older children (9-13) - Adolescents (14-17) - Young adults (18-24) |
| Modality | The *modality* referred to the type of art form employed   - Dance/movement - Literary arts - Media - Music - Theatre/performance - Visual arts, craft, and design - Multimodal, i.e., a combination of more than one art form and/or a mixed modality. |
| Intervention type | Interventions were categorised as either:   - Arts therapy: Interventions that involve a therapeutic relationship within an agreed therapeutic frame delivered by a qualified arts therapist - Not arts therapy: Arts-based interventions that did not meet the criteria for arts therapy, regardless of the intervention’s title or the presence/absence of a qualified arts therapist as a facilitator. |
| Engagement | *Engagement* was classified as either active or receptive:   - Active engagement: Direct participation in arts-based activities (e.g., music-making, singing, dancing, acting, drawing). - Receptive engagement: Observing or receiving arts-based content (e.g., music listening, watching a performance, viewing artwork). |
| Comparator | The *comparator* referred to either an active control or a passive control:   - Active control: Alternative interventions, behavioural interventions or other structured activities. - Passive control: Waiting list, no treatment, or usual care. |
| Number of sessions | *Number of sessions* was categorised as :   - Single session - Two to Twelve sessions - Thirteen or more |
| Risk of Bias | *Risk of Bias* was categorised as:   - Low - Some concerns - High |

## Supplementary Table 3. Removing studies judged high risk of bias.

|  | Number of patients | Number of comparisons | Random effects analysis  Effect size(95% CI) | Number of high ROB comparisons | Random effects analysis  Effect size(95% CI) after removing high risk studies |
| --- | --- | --- | --- | --- | --- |
| **Depression (SMD)**  End of intervention | 1655 | 23 | -1.06(-1.67 to -0.45) | 9 | -0.70(-1.20 to -0.21) |
| **Depression (SMD)**  At least one month after end | 399 | 4 | -0.51(-0.71 to -0.31) | 1 | -0.55(-0.92 to -0.19) |
| **Anxiety (SMD)**  End of intervention | 3902 | 56 | -0.75(-0.94 to -0.57) | 10 | -0.71(-0.90 to -0.52) |
| **Anxiety (SMD)**  At least one month after end | 720 | 7 | -0.67(-1.13 to -0.20) | 2 | -0.85(-1.40 to -0.30) |
| **Wellbeing (SMD)**  End of intervention | 538 | 7 | 0.53(0.26 to 0.80) | 1 | 0.61(-0.24 to 0.97) |
| **QoL (SMD)**  End of intervention | 441 | 10 | 0.54(0.25 to 0.83) | 3 | 0.58(0.19 to 0.97) |
| **Cortisol (SMD)**  End of intervention | 261 | 4 | -0.24(-0.50 to 0.01) | 0 | -0.24(-0.50 to 0.01) |
| **Systolic blood** **pressure (MmHg)**  End of intervention | 693 | 15 | -4.04(-7.3r to -0.75) | 1 | -4.03(-7.64 to -0.41) |
| **Heart rate (BPM)**  End of intervention | 1680 | 29 | -4.53(-6.76 to -2.31) | 2 | -4.41(-6.75 to -2.07) |

## Supplementary Table 4. Summary of overall findings with GRADE Assessment

| **Outcomes** | **Studies measurements** | **SMD effect estimates (95%CI, I²)** | **Certainty of evidence  (GRADE)** | **Plain language summary** |
| --- | --- | --- | --- | --- |
| Depression | Measured by different questionnaires, such as the Beck Depression Inventory (BDI): higher score = higher depression level. Based on SMD data from 1655 participants in 22 studies | SMD= -1.06  ([-1.67 -0.45], 97%) | Very low*  Due to serious inconsistency, serious risk of bias and imprecision. | Arts-based interventions may reduce depression by an average of 4 to 16 points in the BDI scale. |
| Anxiety | Measured by different questionnaires, such as the State–Trait Anxiety Inventory (STAI). 20–80, higher score = higher anxiety level. Based on SMD data from 3898 participants in 48 studies | SMD= -0.75  ([-0.94 -0.57], 87% ) | Very low*  Due to serious inconsistency, serious risk of bias and imprecision | Arts-based interventions may reduce anxiety by an average of 9 to 16 points in the STAI scale |
| Wellbeing | Measured by different questionnaires, such as the Mental Health Continuum-Short Form (MHC-SF).  Higher score = better wellbeing. Based on SMD data from 538 participants in 6 studies. | SMD= 0.53  ( [0.26-0.80], 49%) | Low¶  Due to serious risk of bias and imprecision | Arts-based interventions possibly increase Wellbeing by an average of 3 to 11 points on the MHC-SF scale. |
| Quality of life | Measured by different questionnaires, such as the World Health Organisation Quality Of Life (WHOQOL-BREF) scale.  Higher score= better health-related quality of life. Based on SMD data from 441 participants in 9 studies. | SMD= 0.54  ( [0.25-0.83],441, 53%) | Low¶  Due to risk of bias and imprecision | Arts-based interventions possibly increase quality of life by an average of 3 to 13 points in the WHOQOL-BREF scale. |
| Heart rate | Measured by electronic devices in BPM.  Based on MD data from 1680 participants in 25 studies | MD= -4.54  ([-6.77, -2.31], 77%) | Moderate#  Due to inconsistency | Arts-based interventions possibly reduce Heart rate by an average of 2 BPM to 7 BPM. |
| Blood pressure | Measured by electronic devices in mmHg. Based on MD data from 693 participants in 12 studies | MD= -4.04  ([-7.34, -0.75], 71%) | Moderate#  Due to inconsistency and imprecision | Arts-based interventions possibly reduce Blood pressure by an average of 1 mmHg to 7 mmHg |
| Cortisol | Measured by laboratory test through different types of samples, such as blood (nmol/L). Based on SMD data from 261 participants in 4 studies. | SMD= -0.24  ([-0.50, 0.01], 8%) | Moderate$  Due to serious imprecision | There is no evidence of an effect of art-based interventions on Cortisol |

¶ Although a quite large confidence interval leading to uncertainty in the magnitude of the effect size, all point estimates consistently suggested an increasing effect so we downgraded once for imprecision. However, more than 15% of the studies were at high risk of bias, and therefore, we downgraded twice for risk of bias.

* Although a quite large confidence interval leading to uncertainty in the magnitude of the effect size, all point estimates suggested a reducing effect so we downgraded once for imprecision . We downgraded for serious inconsistency because of the large heterogeneity( I²>70%), and we also downgraded twice due to a serious risk of bias (at 25% of the studies have a high risk).

\# Although a quite large confidence interval leading to uncertainty in the magnitude of the effect size, all point estimates suggested a reducing effect so we downgraded once for imprecision, and due to a high heterogeneity ( I²>70%), we downgraded for inconsistency.

$ We downgraded twice due to serious imprecision as the point estimate suggests a reducing or an increasing effect.

## Supplementary Table 5. Data extraction columns and coding framework used in Elicit

| **Columns** | **Code** |
| --- | --- |
| Author/Year |  |
| Country |  |
| Study Year |  |
| Study Design | RCT, CRCT, Cross-over RCT, Feasibility RCT, Stepped-wedge |
|  | Number of arms |
| Population | Children, adolescents, young adults |
| *Needs/health conditions* | Neurotypical, Neurodivergent, Mental Health, Physical Health, Learning Disability, No apparent needs, other |
| *Age Range / Mean Age* | Mean age (SD) or range |
| Sample Size (N) | N=total, female/male, number randomised per arm and number analysed per arm |
| Setting | Hospital, other clinical settings, education/school, community, online, other |
| Follow-up Period |  |
| Intervention Details |  |
| *Arts Modality* | Music, Dance, Drama, Visual Arts, Literary arts, multimodal |
| *Description of Intervention* |  |
| *Duration/Frequency of Intervention* |  |
| *Facilitator Description* |  |
| *Type of arts intervention* | CAT (specify), arts education, arts intervention with therapeutic intent, other arts-based intervention, self-directed arts participation, other |
| *Engagement type* | Active vs passive |
| Comparators | No treatment, waitlist, usual care, other interventions (specify) |
| List of outcome measures |  |
| Statistical Outcomes | Mean (SD or 95% CI) Post Intervention for both Intervention and comparator, no p-values or interpretation |
| *Timing of outcomes* | Immediately after intervention, first available follow up (minimum 1 month post intervention), latest available follow up (minimum 6 months post intervention) |

**Note on AI-assisted data extraction**
The above table presents the column names and corresponding code used in Elicit for structured data extraction; no free-text or iterative prompts were used. While discrepancies between AI-assisted extraction and manual review were not formally quantified, factual study characteristics (e.g., sample size, region, study design, outcome measures, and intervention descriptions) were generally extracted with high accuracy, whereas more conceptual classifications (e.g., setting definitions, arts modality, health condition categories, type of intervention, and engagement type) and specific statistical outcomes required manual verification and refinement. All AI-generated outputs were reviewed and finalised by two reviewers to ensure accuracy and consistency. A screenshot of the column settings used in Elicit is provided for transparency.

## Supplementary Table 6. List of included studies

|  | **First author, year** | **Title** |
| --- | --- | --- |
| 1 | Abbasi, 2021 | The efficacy of Little Lovely Dentist, dental song, and tell-show-do techniques in alleviating dental anxiety in paediatric patients: A clinical trial |
| 2 | Abd-Elshafy, 2015 | Not all sounds have negative effects on children undergoing cardiac surgery |
| 3 | Abdi, 2025 | Effect of play therapy and storytelling on the anxiety level of hospitalized children: A randomized controlled trial |
| 4 | Abdulah, 2018 | Effectiveness of group art therapy on quality of life in paediatric patients with cancer: A randomized controlled trial |
| 5 | Agres, 2025 | The impact of performing arts on mental health, social connection, and creativity in university students: A randomised controlled trial |
| 6 | Akard, 2021 | Randomized clinical trial of a legacy intervention for quality of life in children with advanced cancer |
| 7 | Alemdar, 2023 | Impact of music therapy and hand massage in the pediatric intensive care unit on pain, fear and stress: randomized controlled trial |
| 8 | Alkan, 2021 | The effects of basic photography education on quality of life, self-esteem, life satisfaction and moods in children with diplegic cerebral palsy: A randomized controlled study |
| 9 | Bali, 2025 | Assessment of smartphone-based active distraction in association with audioanalgesia for overcoming airotor-related anxiety in children: A randomized controlled trial |
| 10 | Barrera, 2018a | A randomized controlled trial of a group intervention for siblings of children with cancer: Changes in symptoms of anxiety in siblings and caregivers |
|  | Barrera, 2018b | Depression and quality of life in siblings of children with cancer after group intervention participation: A randomized control trial |
| 11 | Bay, 2021 | Effect of music on pediatric endoscopic examinations: A randomized controlled trial. |
| 12 | Beebe, 2010 | A randomized trial to test the effectiveness of art therapy for children with asthma |
| 13 | Betzalel, 2017 | The impact of bibliotherapy superheroes on youth who experience parental absence |
| 14 | Bhusari, 2023 | Comparative evaluation of anxiety level during restorative treatment using no music, monaural beats, and binaural auditory beats as audio distraction behavior guidance technique in children aged 6-12 years: A randomized clinical trial. |
| 15 | Bhusari, 2025 | Binaural auditory beats vs music of choice as audio distraction behaviour guidance technique among children: A randomized controlled trial. |
| 16 | Bogaert, 2024 | Recalling and anticipating positive events to improve the positive affect and mental health of adolescents: A cluster randomized controlled trial in secondary schools |
| 17 | Bulut, 2020 | The effect of music therapy, hand massage, and kaleidoscope usage on postoperative nausea and vomiting, pain, fear, and stress in children: A randomized controlled trial |
| 18 | Burns, 2009 | Exploring the feasibility of a therapeutic music video intervention in adolescents and young adults during stem-cell transplantation |
| 19 | Chawla, 2025 | Comparative evaluation of effectiveness among various sensory distractors for reducing dental anxiety in pediatric population aged 6-9 years - A randomized controlled trial. |
| 20 | Chen, 2015 | The effects of Chinese five-element music therapy on nursing students with depressed mood |
| 21 | Chen, 2019 | Effects of preferred music therapy on peer attachment, depression, and salivary cortisol among early adolescents in Taiwan |
| 22 | Cheng, 2023 | Effects of a group-based music imagery program on promoting coping resources among undergraduate students: A pilot randomized controlled trial |
| 23 | Cheraghi, 2021 | A comparison of the effect of visual and auditory distractions on physiological indicators and pain of burn dressing change among 6-12-year-old children: A clinical trial study |
| 24 | Cheung, 2019 | Efficacy of musical training on psychological outcomes and quality of life in Chinese pediatric brain tumor survivors |
| 25 | Choi, 2023 | Art as relaxation for tic disorders: A pilot randomised control study |
| 26 | Çitil, 2024 | The effect of art on premenstrual syndrome symptoms a randomized controlled study |
| 27 | Cleary, 2017 | An aerobic exercise program for young people with cerebral palsy in specialist schools: A phase I randomized controlled trial |
| 28 | Corbett, 2017 | Changes in anxiety following a randomized control trial of a theatre-based intervention for youth with autism spectrum disorder |
| 29 | Corbett, 2023 | Peers, play, and performance to build social salience in autistic youth: A multisite randomized clinical trial |
| 30 | Currie, 2012 | Doing Anger Differently: Two controlled trials of percussion group psychotherapy for adolescent reactive aggression |
| 31 | de la Torre-Luque, 2017 | Effects of preferred relaxing music after acute stress exposure: A randomized controlled trial |
| 32 | Dehghan, 2017 | The effectiveness of dramatic puppet and therapeutic play in anxiety reduction in children undergoing surgery: A randomized clinical trial |
| 33 | Erkut, 2024 | The effect of guided imagery on procedural pain in children: A randomized controlled trial |
| 34 | Forouzandeh, 2020 | The effect of interactive games compared to painting on preoperative anxiety in Iranian children: A randomized clinical trial |
| 35 | Franzoi, 2016 | Music listening for anxiety relief in children in the preoperative period: A randomized clinical trial |
| 36 | Gee, 2019 | Blue Notes: Using songwriting to improve student mental health and wellbeing. a pilot randomised controlled trial |
| 37 | Goktas, 2023 | The effect of visual and/or auditory distraction techniques on children's pain, anxiety and medical fear in invasive procedures: A randomized controlled trial |
| 38 | Gold, 2017 | Group music therapy as a preventive intervention for young people at risk: cluster-randomized trial |
| 39 | Goldbeck, 2012 | A randomized controlled trial of multimodal music therapy for children with anxiety disorders |
| 40 | Gómez Scarpetta, 2012 | Music therapy to control dental anxiety in children with Down syndrome |
| 41 | Gozin, 2022 | Audio-visual storytelling for reducing dental anxiety in Iranian children: A randomized controlled trial |
| 42 | Gulmez, 2023 | Effects of Maqams in music on anxiety: A preliminary, randomized controlled trial |
| 43 | Günay, 2022 | The effects of the activity of making jewelry from beads on the anxiety levels of children with cancer: A randomised controlled study |
| 44 | Gurcan, 2021 | The effectiveness of mandala drawing in reducing psychological symptoms, anxiety and depression in hospitalised adolescents with cancer: A randomised controlled trial |
| 45 | Hides, 2019 | Efficacy and outcomes of a music-based emotion regulation mobile app in distressed young people: randomized controlled trial |
| 46 | Hosseini, 2023 | The effect of mandala therapy on anxiety, pain severity and distress of primary dysmenorrhea: A randomized clinical trial |
| 47 | Irons, 2012 | Singing therapy for young people with cystic fibrosis: A randomized controlled pilot study. |
| 48 | Janthasila, 2023 | Music therapy and aromatherapy on dental anxiety and fear: A randomized controlled trial |
| 49 | Javed, 2021 | Medical clowning: A cost-effective way to reduce stress among children undergoing invasive procedures. |
| 50 | Jeong, 2005 | Dance movement therapy improves emotional responses and modulates neurohormones in adolescents with mild depression |
| 51 | Karakul, 2022 | Effectiveness of music during cardiac catheterization on children's pain, fear, anxiety and vital signs: A randomized, blind controlled trial |
| 52 | Kim, 2024 | Effects of a choral program combining wind instrument performance and breathing training on respiratory function, stress, and quality of life in adolescents: A randomized controlled trial |
| 53 | Kirca, 2022 | Effects of music medicine on premenstrual symptoms levels and quality of life: A randomized controlled trial |
| 54 | Koenig, 2013 | Specific music therapy techniques in the treatment of primary headache disorders in adolescents: A randomized attention-placebo-controlled trial |
| 55 | Lai, 2022 | Telehealth movement-to-music to increase physical activity participation among adolescents with cerebral palsy: pilot randomized controlled trial |
| 56 | Lee, 2016 | Effects of music therapy on the cardiovascular and autonomic nervous system in stress-induced university students: A randomized controlled trial |
| 57 | Lindor, 2023 | The feasibility and acceptability of AllPlay Dance for autistic children: A pilot randomised controlled trial |
| 58 | Malakoutikhah, 2020 | The effect of different genres of music and silence on relaxation and anxiety: A randomized controlled trial. |
|  | Malakoutikhah, 2023 | A randomized controlled trial on the effects of different music genres on physiologic parameters and emotion |
| 59 | Martin-Saavedra, 2020 | The effects of music listening on the management of pain in primary dysmenorrhea: A randomized controlled clinical trial. |
| 60 | Mir, 2021 | Relaxing music reduces blood pressure and heart rate among pre-hypertensive young adults: A randomized control trial |
| 61 | Mittal, 2025 | Impact of dentistry modeling story on dental anxiety in 6-8-year-old children of industrial workers: A randomized controlled trial. |
| 62 | Mohanasundari, 2023 | Comparison of music medicine intervention and visual imagery techniques and conventional treatment alone on preoperative anxiety among children undergoing surgery - A Randomized Control Trial |
| 63 | Mojahed, 2021 | Effectiveness of group psychodrama on aggression and social anxiety of children with attention-deficit/hyperactivity disorder: A randomized clinical trial |
| 64 | Moula, 2020 | An investigation of the effectiveness of arts therapies interventions on measures of quality of life and wellbeing: A pilot randomized Controlled Study in Primary Schools |
| 65 | Muragod, 2024 | Effect of music therapy versus guided imagery on healthcare professional students with stress and anxiety: A randomized clinical trial. |
| 66 | Nahir, 2024 | Evaluation of the role of music and Bach flower remedies in the management of anxious pediatric dental patients: A randomised clinical trial |
| 67 | Nuvvula, 2015 | Effect of audiovisual distraction with 3D video glasses on dental anxiety of children experiencing administration of local analgesia: A randomised clinical trial. |
| 68 | Osborn, 2023 | An arts-literacy intervention for adolescent depression and anxiety symptoms: outcomes of a randomised controlled trial of Pre-Texts with Kenyan adolescents |
| 69 | Ozturk, 2022 | The effect of mindfulness-based mandala activity on anxiety and spiritual well-being levels of senior nursing students: A randomized controlled study |
| 70 | Pang, 2025 | The effectiveness of Chinese instrumental music embedded with binaural beats in relieving anxiety related to academic stress among undergraduates: A randomized controlled trial |
| 71 | Piasai, 2018 | A randomized control trial of guided-imagination and drawing-storytelling in children with cancer. |
| 72 | Porter, 2017 | Music therapy for children and adolescents with behavioural and emotional problems: A randomised controlled trial |
| 73 | Rafatjou, 2021 | Evaluation effect of color in dental office and dentist's uniform while using two different distraction techniques on injection anxiety of 6-9 years' old children referring to Hamedan Dental School: Randomized clinical trial. |
| 74 | Rezazadeh, 2020 | Effects of art therapy program on anxiety and depression among 6-12-year-old burned children |
| 75 | Rodríguez-Jiménez, 2022 | Embodied learning for well-being, self-awareness, and stress regulation: A randomized trial with engineering students using a mixed-method approach |
| 76 | Sadeghi, 2023 | Which audio distraction technique is more effective for reduction the pain and anxiety of pediatric dental patients; music or kids-story? A randomized split-mouth crossover clinical trial |
| 77 | Selçuk, 2022 | The effect of dark chocolate and music on pain and anxiety in young women with primary dysmenorrhea: Randomized controlled trial |
| 78 | Shahrbabaki, 2025 | Effectiveness of mandala coloring intervention on children's preoperative anxiety: A randomized clinical trial study in Iran |
| 79 | Sharda, 2018 | Music improves social communication and auditory–motor connectivity in children with autism |
| 80 | Shawq, 2025 | The effects of music interventions on children's pain severity and anxiety during dressing changes in Iraq: A randomized controlled trial. |
| 81 | Sheriff, 2023 | A co-produced online cultural experience compared to a typical museum website for mental health in people aged 16-24: A proof-of-principle randomised controlled trial |
| 82 | Siegel, 2016 | Expressive arts therapy with hospitalized children: A pilot study of co-creating healing sock creatures© |
| 83 | Song, 2021 | Effects of the Healing Beats Program among university students after exposure to a source of psychological stress: A randomized control trial |
| 84 | Soqia, 2025 | Evaluating the efficacy of a 5-min music listening intervention for state anxiety reduction in college students: A randomized controlled trial |
| 85 | Stice, 2007 | Randomized trial of a brief depression prevention program: An elusive search for a psychosocial placebo control condition |
| 86 | Stinley, 2015 | Creating mandalas for the management of acute pain symptoms in pediatric patients |
| 87 | Tan, 2021 | Effects of sandplay therapy in reducing emotional and behavioural problems in school‐age children with chronic diseases: A randomized controlled trial. |
| 88 | Thanh, 2010 | Music therapy to reduce pain and anxiety in children with cancer undergoing lumbar puncture: A randomized clinical trial |
| 89 | Ubrangala, 2022 | Effect of Indian music as an auditory stimulus on physiological measures of stress, anxiety, cardiovascular and autonomic responses in humans-a randomized controlled trial |
| 90 | Valizadeh, 2022 | The effects of storytelling on anxiety and sleep in hospitalized children with fracture: A randomized clinical trial |
| 91 | van der Heijden, 2019 | Children listening to music or watching cartoons during ER procedures: A RCT |
| 92 | Wang, 2023 | Effectiveness of two distraction strategies in reducing preoperative anxiety in children in China: A randomized controlled trial |
| 93 | Xu, 2024 | Assessing dance movement therapy in Chinese undergraduates with depression and anxiety: An initial randomized controlled trial |
| 94 | Yan, 2019 | School bullying among left-behind children: The efficacy of art therapy on reducing bullying victimization |
| 95 | Yi, 2024 | Cognitive behavior, art, and music therapies intervention for treating the depression of children: A randomized control trial |
| 96 | Yildirim, 2025 | Effect of toys made from medical materials on anxiety among hospitalized children: A randomized controlled trial. |
| 97 | Yu, 2009 | Effects of music on anxiety and pain in children with cerebral palsy receiving acupuncture: A randomized controlled trial |
| 98 | Yücel, 2024 | The effect of listening to music and foot reflexology on nausea, pain and anxiety in children during perioperative period: A randomized controlled study |
| 99 | Zamanifard, 2022 | The effects of virtual directed painting therapy on anxiety, depression, and self-efficacy of children with type 1 diabetes: A randomized controlled clinical trial. |
| 100 | Zhang, 2022 | Effect of group impromptu music therapy on emotional regulation and depressive symptoms of college students: A randomized controlled study |

## Supplementary Table 7. List of excluded studies with reasons

|  | **First author, year** | **Title** |
| --- | --- | --- |
| Wrong population n=125 | | |
| 1 | Abbasi, 2023 | Distraction techniques for post-operative paediatric patients in post anesthesia care unit (PACU): A randomized control trial |
| 2 | Abessa, 2019 | Effect of play-based family-centered psychomotor/psychosocial stimulation on the development of severely acutely malnourished children under six in a low-income setting: A randomized controlled trial. |
| 3 | Aboalshamat, 2023 | Storytelling as innovative method to improve the recognition of teledentistry among adults: A randomized controlled trial. |
| 4 | Adam, 2025 | Effect of short, animated storytelling, conveying the lived experiences of a mother vs a father of a trans child, on transphobia among US parents: A randomized controlled trial |
| 5 | Afshar, 2023 | What effects can expressive writing have on sexual dysfunction in women with multiple sclerosis? a randomized controlled trial |
| 6 | Albornoz, 2011 | The effects of group improvisational music therapy on depression in adolescents and adults with substance abuse: A randomized controlled trial |
| 7 | Almedhesh, 2022 | The effect of virtual reality on anxiety, stress, and hemodynamic parameters during cesarean section A randomized controlled clinical trial |
| 8 | Amsalem, 2025 | Effect of a short, animated storytelling video on transphobia among us parents: randomized controlled trial |
| 9 | An, 2024 | A randomized controlled study in young adults to compare changes in creativity after a new mind-body based intervention Quadrato Motor Training |
| 10 | Aravena, 2020 | Effect of music at 432 Hz and 440 Hz on dental anxiety and salivary cortisol levels in patients undergoing tooth extraction: A randomized clinical trial. |
| 11 | Armstrong, 2021 | Randomised controlled trial of a therapeutic playgroup for children with developmental delays. |
| 12 | Badr, 2023 | Effect of Benson's relaxation technique versus music intervention on physiological parameters and stress of children with thalassemia during blood transfusions: A randomized controlled trial |
| 13 | Bakhshandeh, 2024 | The effect of music therapy on the pain level of 3 to 6-year-old children undergoing wound repair by suturing and their parents' anxiety in the emergency department: A randomized controlled trial. |
| 14 | Baltacı, 2021 | The effect of lullaby intervention on anxiety and prenatal attachment in women with high-risk pregnancy: A randomized controlled study |
| 15 | Bartakke, 2025 | Comparison of the efficacy of aromatherapy and bubble breath play therapy in reducing dental anxiety in children: A randomized clinical trial. |
| 16 | Bauer, 2010 | Alleviating distress during antepartum hospitalization: A randomized controlled trial of music and recreation therapy. |
| 17 | Berbel, 2007 | [Music versus diazepam to reduce preoperative anxiety: A randomized controlled clinical trial]. |
| 18 | Bingöl, 2022 | Psychodrama as a new intervention for reducing fear of childbirth: A randomised controlled trial |
| 19 | Bodner, 2012 | Reduction of seizure occurrence from exposure to auditory stimulation in individuals with neurological handicaps: A randomized controlled trial. |
| 20 | Brockmeyer, 2021 | Cognitive remediation therapy in anorexia nervosa-a randomized clinical trial |
| 21 | Bugos, 2022 | Multimodal music training enhances executive functions in children: Results of a randomized controlled trial |
| 22 | Chen, 2016 | Randomized trial of group music therapy with Chinese prisoners: impact on anxiety, depression, and self-esteem. |
| 23 | Chien, 2016 | A randomized controlled trial of clinician-supported problem-solving bibliotherapy for family caregivers of people with first-episode psychosis. |
| 24 | Contreras, 2024 | Music in academia for undergraduate nursing students: A randomized controlled study |
| 25 | Corbett, 2023 | Investigating social competence in a pilot randomized clinical trial of a theatre-based intervention enhanced for adults with autism spectrum disorder |
| 26 | Craddock, 2025 | "My body is amazing from the bottom to the top" - An RCT study testing two positive body image media micro-interventions for young children aged 4-6 years |
| 27 | Daihimfar, 2024 | A comparison of the effects of acupressure and music on venipuncture pain intensity in children: A randomized controlled clinical trial |
| 28 | Deichman, 2025 | Gratitude training for promoting subjective well-being: A randomized controlled trial comparing journaling to a personalized menu approach |
| 29 | Demir, 2023 | The effect of auditory stimuli on the pain and physiological parameters of children on mechanical ventilation during aspiration procedure: A randomized controlled trial |
| 30 | Di Nasso, 2016 | Influences of 432 hz music on the perception of anxiety during endodontic treatment: A randomized controlled clinical trial. |
| 31 | Dixit, 2020 | Comparison of the effectiveness of Bach flower therapy and music therapy on dental anxiety in pediatric patients: A randomized controlled study. |
| 32 | Dixon, 2019 | The effect of distraction during labor induction on timing of analgesia request: A randomized clinical trial. |
| 33 | Drzymalski, 2023 | The effect of Mozart music on patient satisfaction during caesarean delivery: A randomised controlled trial |
| 34 | E Aksoy, 2024 | The effect of music therapy on non-stress test results and anxiety levels in high-risk pregnant women: A randomized controlled trial. |
| 35 | Ebrahimi, 2019 | The effect of filial therapy on depressive symptoms of children with cancer and their mother's depression, anxiety, and stress: A randomized controlled trial |
| 36 | El Rafihi-Ferreira, 2018 | Brief treatment for nighttime fears and co-sleeping problems: A randomized clinical trial |
| 37 | Eskici İlgin, 2024 | The effect of movie-watching on depression, anxiety and stress in hospitalised patients with COVID-19: A randomized controlled study |
| 38 | Favaretti, 2023 | Participant engagement with a short, wordless, animated video on COVID-19 prevention: A multi-site randomized trial. |
| 39 | Feldman, 2016 | Psychological treatment of comorbid asthma and panic disorder in Latino adults: Results from a randomized controlled trial. |
| 40 | Ferraz, 2021 | Analgesic effect of music during wound care among patients with diaphyseal tibial fractures: Randomized controlled trial |
| 41 | Firmeza, 2017 | Control of anxiety through music in a head and neck outpatient clinic: A randomized clinical trial |
| 42 | Furmark, 2009 | Guided and unguided self-help for social anxiety disorder: randomised controlled trial. |
| 43 | Gallego-Gómez, 2020 | Effectiveness of music therapy and progressive muscle relaxation in reducing stress before exams and improving academic performance in Nursing students: A randomized trial |
| 44 | Gauthier, 2024 | The effect of music therapy and breathing exercise on anxiety and pain in patients undergoing coronary angiography: A randomized controlled study. |
| 45 | Gezginci, Iyigun, Kibar,, 2018 | Three distraction methods for pain reduction during cystoscopy: A randomized controlled trial evaluating the effects on pain, anxiety, and satisfaction. |
| 46 | Gezginci, 2018 | Comparison of two different distraction methods affecting the level of pain and anxiety during extracorporeal shock wave lithotripsy: A randomized controlled trial. |
| 47 | Ghezeljeh, 2016 | The effects of music intervention on background pain and anxiety in burn patients: randomized controlled clinical trial. |
| 48 | Ghorbani, 2025 | Evaluating the effectiveness of mandala coloring on post-operative anxiety in mothers of children undergoing congenital anomaly surgery: A randomized controlled clinical trial. |
| 49 | Guerra, 2021 | Music Use for Sedation in Critically ill Children (MUSiCC trial): A pilot randomized controlled trial |
| 50 | Guerrero, 2012 | Music as an auxiliary analgesic during first trimester surgical abortion: A randomized controlled trial. |
| 51 | Hartling, 2013 | Music to reduce pain and distress in the pediatric emergency department a randomized clinical trial |
| 52 | He, 2022 | [Effect of parent-child cooperative music therapy on children with autism spectrum disorder and their mothers: A prospective randomized controlled study]. |
| 53 | He, 2024 | Effects of Mozart-Orff parent-child music therapy among mothers and their preschool children with autism spectrum disorder: A mixed-methods randomised controlled trial |
| 54 | Hedman, 2016 | Exposure-based cognitive-behavioural therapy via the internet and as bibliotherapy for somatic symptom disorder and illness anxiety disorder: randomised controlled trial. |
| 55 | Herlekar, 2018 | TRAIT level of perceived anxiety and effect of instrumental background music after induced stress test on STATE level of perceived anxiety in students from different ethnic groups-A randomized control trial |
| 56 | Herlekar, 2017 | Stress perceived and its effect on concentration in first year medical students of different ethnicities. Can instrumental background music act as a common coping strategy? A randomised controlled trial |
| 57 | Ho, 2016 | Happy Family Kitchen II: A cluster randomized controlled trial of a community-based positive psychology family intervention for subjective happiness and health-related quality of life in Hong Kong. |
| 58 | Hoegholt, 2024 | Music and sleep hygiene interventions for pregnancy-related insomnia: An online randomized controlled trial |
| 59 | Huang, 2017 | Effects of music and music video interventions on sleep quality: A randomized controlled trial in adults with sleep disturbances. |
| 60 | DRUMMOND, 2018 | Rapid induction analgesia for capsaicin-induced pain in university students: A randomized, Controlled Trial |
| 61 | Jiang, 2024 | Personal versus therapist perioperative music intervention: A randomized controlled trial. |
| 62 | Jin, 2021 | Self-produced audio-visual animation introduction alleviates preoperative anxiety in pediatric strabismus surgery: A randomized controlled study |
| 63 | Kantor, 2022 | Effect of low frequency sound vibration on acute stress response in university students-Pilot randomized controlled trial |
| 64 | Karaca, 2021 | The effect of music-moving toys to reduce fear and anxiety in preschool children undergoing intravenous insertion in a pediatric emergency department: A randomized clinical trial |
| 65 | Kaya, 2025 | The effect of music recital on labor anxiety and satisfaction: A randomized controlled study. |
| 66 | Kim, 2008 | The effects of improvisational music therapy on joint attention behaviors in autistic children: A randomized controlled study |
| 67 | Koç, 2024 | Effect of a musical toy used during peripheral venous access on children's pain, fear and parental satisfaction: Randomized controlled trial |
| 68 | Kuo, 2018 | Distraction-based interventions for children undergoing venipuncture procedures: A randomized controlled study. |
| 69 | Kupeli, 2020 | Comparing different music genres in decreasing dental anxiety in young adults who underwent third molar surgery in Turkey: randomized controlled trial |
| 70 | Lee, 2017 | Comparing effects between music intervention and aromatherapy on anxiety of patients undergoing mechanical ventilation in the intensive care unit: A randomized controlled trial. |
| 71 | Lee, 2023 | Effect of a virtual reality environment using a domed ceiling screen on procedural pain during intravenous placement in young children: A randomized clinical trial. |
| 72 | Lin, 2025 | Effects of a WeChat-based parent-child creative art therapy for mothers of children with autism spectrum disorder: A pilot randomized controlled trial |
| 73 | Liu, 2023 | Effects of Parent-Child Sandplay Therapy for preschool children with autism spectrum disorder and their mothers: A randomized controlled trial. |
| 74 | Lohr, 2023 | Stories for change protocol: A randomized controlled trial of a digital storytelling intervention for Hispanic/Latino individuals with type 2 diabetes. |
| 75 | Macuncu, 2025 | The effect of storytelling and painting on children's fear level and parental health care satisfaction in children aged 3-6 years administered inhaler medication: Randomized controlled study |
| 76 | McCann, 2013 | A randomized controlled trial of bibliotherapy for carers of young people with first-episode psychosis |
| 77 | Mehr, 2013 | Two randomized trials provide no consistent evidence for nonmusical cognitive benefits of brief preschool music enrichment |
| 78 | Meiri, 2016 | The effect of medical clowning on reducing pain, crying, and anxiety in children aged 2-10 years old undergoing venous blood drawing-a randomized controlled study |
| 79 | Mietzsch, 2020 | Classical but not rap music significantly improves transferability and long-term acquisition of laparoscopic suturing skills: A randomized controlled trial |
| 80 | Mounier, 2024 | Music therapy during basic daily care in critically ill children: A randomized crossover clinical trial |
| 81 | Muzzi, 2021 | Effects of intraoperative auditory stimulation on pain and agitation on awakening after pediatric adenotonsillectomy a randomized clinical trial |
| 82 | Nyenhuis, 2013 | The efficacy of minimal contact interventions for acute tinnitus: A randomised controlled study. |
| 83 | Orak, n.d. | [Effects of music therapy on pain and oxidative stress in oocyte pick-up: A randomized clinical trial]. |
| 84 | Ortega, 2019 | Music therapy for pain and anxiety management in nasal bone fracture reduction: randomized controlled clinical trial. |
| 85 | Osman, 2014 | Interventions to reduce postpartum stress in first-time mothers: A randomized-controlled trial. |
| 86 | Paprad, 2019 | The effect of Mozart K. 448 music on interictal epileptiform discharges in children with epilepsy: A randomized controlled trial: A pilot study |
| 87 | Perkins, 2023 | Online songwriting reduces loneliness and postnatal depression and enhances social connectedness in women with young babies: randomised controlled trial |
| 88 | Pinniger R, 2012 | Argentine tango dance compared to mindfulness meditation and a waiting-list control: A randomised trial for treating depression. |
| 89 | Preethy, 2020 | Effect of music on the vital signs and behaviour of children during the recovery period after general anaesthesia procedure-a randomized controlled trial |
| 90 | Raglio, 2020 | Machine learning techniques to predict the effectiveness of music therapy: A randomized controlled trial. |
| 91 | Rennick, 2018 | A pilot randomized controlled trial of an intervention to promote psychological well-being in critically ill children: soothing through touch, reading, and music |
| 92 | Robb, 2017 | Pilot randomized trial of active music engagement intervention parent delivery for young children with cancer |
| 93 | Sahebalam, 2020 | Comparison of the efficacy of Jilo animation approach versus conventional tell-show-do (tsd) technique on cooperation and anxiety levels of children during dental practice: A randomized controlled clinical trials. |
| 94 | Salawati Ghasemi, 2021 | Comparison of the effects of Hugo's point massage and play on IV-line placement pain in children: A randomized clinical trial. |
| 95 | Sari, 2019 | A randomized controlled trial to test efficacy of digital enhancements of storybooks in support of narrative comprehension and word learning |
| 96 | Sezici, 2017 | Use of play therapy in nursing process: A prospective randomized controlled study |
| 97 | Sim, 2025 | Effectiveness of a universal film intervention in reducing violence against children and increasing positive parenting among migrant and displaced caregivers from Myanmar: A community-based cluster randomised trial |
| 98 | Simavli, 2014 | Effect of music on labor pain relief, anxiety level and postpartum analgesic requirement: A randomized controlled clinical trial. |
| 99 | Son, 2019 | Effects of aromatherapy combined with music therapy on anxiety, stress, and fundamental nursing skills in nursing students: A randomized controlled trial |
| 100 | Stein, 2016 | Clinical trial on tonal tinnitus with tailor-made notched music training. |
| 101 | Strohbehn, 2020 | Visual arts in the clinical clerkship: A pilot cluster-randomized, controlled trial |
| 102 | Sökmen, 2024 | The effect of music on nonstress test and pregnant women's satisfaction: A randomized controlled trial. |
| 103 | TIME-A Study Team, Bieleninik,, 2017 | Effects of improvisational music therapy vs enhanced standard care on symptom severity among children with autism spectrum disorder the TIME-A randomized clinical trial |
| 104 | TIME-A Study Team, Crawford,, 2017 | International multicentre randomised controlled trial of improvisational music therapy for children with autism spectrum disorder: TIME-A study |
| 105 | Tan, 2022 | Effect of a popular web drama video series on HIV and other sexually transmitted infection testing among gay, bisexual, and other men who have sex with men in singapore: community-based, pragmatic, randomized controlled trial. |
| 106 | Tao, 2024 | The effects of combining visual-auditory stimuli with exercise on short-term affect improvement: A randomized controlled trial. |
| 107 | Thompson, 2013 | Family-centred music therapy to promote social engagement in young children with severe autism spectrum disorder: A randomized controlled study |
| 108 | Toker, 2017 | Effect of Turkish classical music on prenatal anxiety and satisfaction: A randomized controlled trial in pregnant women with pre-eclampsia |
| 109 | Tolunay, 2018 | Rhythm and orthopedics: The effect of music therapy in cast room procedures, a prospective clinical trial. |
| 110 | Tuncay, 2023 | The effect of nursing interventions with therapeutic play and video animations prepared with psychodrama technique in reducing fear, anxiety, and pain of children at male circumcision: A randomized controlled study |
| 111 | Uğraş, 2018 | The effect of different types of music on patients' preoperative anxiety: A randomized controlled trial. |
| 112 | Vianna, n.d. | Music therapy may increase breastfeeding rates among mothers of premature newborns: A randomized controlled trial. |
| 113 | Wagner, 2013 | Internet-delivered cognitive-behavioural therapy v. conventional guided self-help for bulimia nervosa: long-term evaluation of a randomised controlled trial. |
| 114 | Weisleder, 2018 | Reading aloud and child development: A cluster-randomized trial in Brazil. |
| 115 | Winters, 2025 | Debunking COVID-19 vaccine misinformation with an audio drama in Ghana, a randomized control trial |
| 116 | Yalcin, 2024 | The effect of a stress and anxiety coping programme on objective structured clinical exam performance in medical students, a randomised clinical trial |
| 117 | Yaz, 2024 | The effect of vibrating cold application and puppet use on pain and fear during phlebotomy in children: A randomized controlled study |
| 118 | Yinger, 2016 | Music therapy as procedural support for young children undergoing immunizations: A randomized controlled study |
| 119 | Zhang, 2020 | Impact of orally administered tramadol combined with self-selected music on adult outpatients with burns undergoing dressing change: A randomized controlled trial. |
| 120 | Zheng, 2025 | Effects of digital picture books on young children undergoing cardiac catheterisation and their family caregivers: A randomised controlled Trial |
| 121 | Zhou, 2025 | A randomized controlled trial of the efficacy of music therapy on the social skills of children with autism spectrum disorder |
| 122 | da Silva, 2021 | Effects of music on the anxiety of blood donors: randomized clinical trial |
| 123 | d’Arma, 2023 | A cultural training for the improvement of cognitive and affective Theory of Mind in people with Multiple Sclerosis: A pilot randomized controlled study |
| 124 | van der Heijden, 2018 | Can live music therapy reduce distress and pain in children with burns after wound care procedures? A randomized controlled trial |
| 125 | Çatalgöl, 2022 | The effects of music therapy applied to pregnant women on maternal, fetal, and neonatal results: A randomized controlled study. |
| Wrong intervention n=81 | | |
| 1 | Agboeze, 2020 | Efficacy of music-based cognitive behavior therapy on the management of test-taking behavior of children in basic science using a randomized trial group: Implication for community development |
| 2 | Aksoy, 2024 | A randomized controlled trial on the effect of music and white noise listening on anxiety and vital signs during intramuscular injection skill learning |
| 3 | Al-Khotani, 2016 | Effects of audiovisual distraction on children's behaviour during dental treatment: A randomized controlled clinical trial |
| 4 | Alagesan, 2011 | Effect of play therapy in children with attention deficit hyperactivity disorder -- a single blinded randomized controlled study. |
| 5 | Aventin, 2019 | Acceptability of an interactive film-based intervention targeting adolescent boys to prevent sexual risk-taking: findings from the JACK cluster randomised controlled trial process evaluation |
| 6 | Azizi, 2020 | Effect of cognitive-behavioral play therapy on working memory, short-term memory and sustained attention among school-aged children with specific learning disorder: A preliminary randomized controlled clinical trial |
| 7 | Bharti, 2016 | MRI Customized play therapy in children reduces the need for sedation - a randomized controlled trial |
| 8 | Calero, 2017 | Can arts-based interventions enhance labor market outcomes among youth? Evidence from a randomized trial in Rio de Janeiro |
| 9 | Chan, 2024 | Impact of child-centered play therapy intervention on children with autism reflected by brain EEG activity: A randomized controlled trial |
| 10 | Chan, 2025 | Association between changes in EEG alpha power and behavioural outcome in autistic children induced by child-centred play therapy: A randomised controlled trial |
| 11 | Chhillar, 2020 | An RCT on Effectiveness of play therapy on anxiety among hospitalized children in selected hospital, Greater Noida |
| 12 | Eyuboglu, 2020 | Effect of music therapy on nursing students' first objective structured clinical exams, anxiety levels and vital signs: A randomized controlled study |
| 13 | Ezegbe, 2019 | Rational emotive digital storytelling therapy for improving HIV/AIDS Knowledge and risk perception among schoolchildren: A group randomized trial |
| 14 | Fazlollahi, 2022 | The effects of meaning-centered play on spiritual sensitivity of children: A randomized controlled trial. |
| 15 | Gebhart, 2020 | Distraction-focused interventions on examination stress in nursing students: Effects on psychological stress and biomarker levels. A randomized controlled trial |
| 16 | Gold, 2021 | Effect of an immersive virtual reality intervention on pain and anxiety associated with peripheral intravenous catheter placement in the pediatric setting a randomized clinical trial |
| 17 | Goyel, 2022 | Evaluation of different pre-treatment behaviour modification techniques in 4-7-year olds: A randomised controlled trial. |
| 18 | Guerra, 2024 | Effect of three group interventions on psychosocial functioning in adolescents exposed to interpersonal violence in Chile: A pilot clinical trial |
| 19 | Hashimoto, 2020 | Video glasses reduce preoperative anxiety compared with portable multimedia player in children: A randomized controlled trial |
| 20 | Hazlett-Steven, 2017 | Effectiveness of mindfulness-based stress reduction bibliotherapy: A preliminary randomized controlled trial |
| 21 | He, 2015 | Therapeutic play intervention on children's perioperative anxiety, negative emotional manifestation and postoperative pain: A randomized controlled trial. |
| 22 | Herlekar, 2019 | Effect of classical instrumental music on successive divided attention tests in Indian and Malaysian first year medical students - A randomized control trial |
| 23 | Hsu, 2022 | Effectiveness of virtual reality interactive play for children during intravenous placement: A randomized controlled trial |
| 24 | Inangil, Sendir,, 2020 | The effect of music therapy and progressive muscle relaxation exercise on anxiety before the first clinical practice in nursing students: A randomized controlled study |
| 25 | Inangil, Vural,, 2020 | Effectiveness of music therapy and emotional freedom technique on test anxiety in Turkish nursing students: A randomised controlled trial |
| 26 | Jibb, 2018 | Using the MEDiPORT humanoid robot to reduce procedural pain and distress in children with cancer: A pilot randomized controlled trial |
| 27 | Karmaliani, 2020 | Right To Play's intervention to reduce peer violence among children in public schools in Pakistan: A cluster-randomized controlled trial. |
| 28 | Kent, 2021 | Can I learn to play? randomized control trial to assess effectiveness of a peer-mediated intervention to improve play in children with autism spectrum disorder. |
| 29 | Kim, 2022 | The efficacy of a forgiveness bibliotherapy: A randomized controlled trial with nursing students |
| 30 | Kooraki, 2023 | Effectiveness of cognitive behavioral psychodrama group therapy in treating self- focused attention and social anxiety of adolescent girls: A randomized clinical trial |
| 31 | Kumar, 2019 | Perioperative anxiety and stress in children undergoing congenital cardiac surgery and their parents: effect of brief intervention-a randomized control trial. |
| 32 | Kunikullaya U., 2022 | Temporal changes in electroencephalographic power spectrum on passive listening to three selected melodic scales of Indian music on healthy young individuals A randomized controlled trial |
| 33 | Lai, 2024 | Application of remodeled glove puppetry for children with developmental disabilities: A randomized controlled trial |
| 34 | Lee, 2011 | A randomized trial of computer-based communications using imagery and text information to alter representations of heart disease risk and motivate protective behaviour |
| 35 | Lee, 2018 | Using narrative intervention for HPV vaccine behavior change among Khmer mothers and daughters: A pilot RCT to examine feasibility, acceptability, and preliminary effectiveness. |
| 36 | Li, 2008 | Effectiveness and appropriateness of therapeutic play intervention in preparing children for surgery: A randomized controlled trial study. |
| 37 | Li, 2014 | Effect of therapeutic play on pre- and post-operative anxiety and emotional responses in Hong Kong Chinese children: A randomised controlled trial. |
| 38 | Liu, 2022 | Using AI chatbots to provide self-help depression interventions for university students: A randomized trial of effectiveness |
| 39 | McMahon, 2015 | A randomized controlled trial of a multi-dose bystander intervention program using peer education theater |
| 40 | Mentes, 2025 | Effects of a nurse-led play-based internet addiction prevention program in early adolescents: A randomized controlled study |
| 41 | Mete, 2024 | The impact of digital storytelling for children during paediatric day surgery on anxiety and negative emotional behaviors: Randomized controlled trial |
| 42 | Mirzaie, 2019 | Comparison of the effect of Filial and Adlerian play therapy on attention and hyperactivity of children with attention deficit hyperactivity disorder: A randomized clinical trial |
| 43 | Mohan, 2015 | The effect of entonox, play therapy and a combination on pain relief in children: A randomized controlled trial |
| 44 | Moldovan, 2013 | Cognitive bibliotherapy for mild depressive symptomatology: randomized clinical trial of efficacy and mechanisms of change |
| 45 | Müller, 2019 | Effect of a multidimensional physical activity intervention on body mass index, skinfolds and fitness in South African children: results from a cluster-randomised controlled trial |
| 46 | Norris, 2013 | Randomized trial of a peer resistance skill-building game for Hispanic early adolescent girls |
| 47 | Obiweluozo, 2021 | Impact of cognitive behavioural play therapy on social anxiety among school children with stuttering deficit A cluster randomised trial with three months follow-up |
| 48 | Park, 2022 | Effects of rhythm step training on physical and cognitive functions in adolescents: A prospective randomized controlled trial |
| 49 | Pearson, 2021 | Thriving in adversity: Do life skills programs work for developing world children? A pragmatic randomized controlled trial |
| 50 | Petrusic, 2022 | Teaching gymnastics elements in elementary school with placebo-effect digital games: cluster-randomised controlled trial |
| 51 | Potasz, 2013 | Effect of play activities on hospitalized children's stress: A randomized clinical trial. |
| 52 | Rapee, 2006 | Bibliotherapy for children with anxiety disorders using written materials for parents: A randomized controlled trial |
| 53 | Ray, 2022 | Child-centered play therapy and adverse childhood experiences: A randomized controlled trial |
| 54 | Rutkowski, 2024 | Training using a commercial immersive virtual reality system on hand-eye coordination and reaction time in students: A randomized controlled trial |
| 55 | Saha, 2023 | Effect of mind sound resonance technique on selected psycho-emotional well-being parameters in secondary school students: A randomised controlled trial |
| 56 | Schottelkorb, 2012 | Treatment for childhood refugee trauma: A randomized, controlled trial |
| 57 | Seale, 2022 | GROW Zambia: A pilot cluster-randomized trial of a spiritually-based character strengths training curriculum to enhance resilience among Zambian youth |
| 58 | Sekhavatpour, 2019 | The effect of storytelling on anxiety and behavioral disorders in children undergoing surgery: A randomized controlled trial. |
| 59 | Sharififard, 2020 | A music- and game-based oral health education for visually impaired school children; multilevel analysis of a cluster randomized controlled trial |
| 60 | Shayganfard, 2024 | Effectiveness of cognitive-behavioral group play therapy on anxiety-based school refusal and behavioral problems in elementary school boys: A preliminary randomized controlled trial |
| 61 | Shrimahalakshmi, 2025 | Effect of bubble breath play therapy in anxiety levels and pain perception in children receiving local anesthesia: A randomized clinical trial. |
| 62 | Siu, 2023 | Evaluating two play-based approaches for children and carers in residential settings: A mixed-method, randomized control study |
| 63 | Soto-Ruiz, 2025 | Reducing medical fear among children using simulated health care spaces: A randomized controlled trial |
| 64 | Stice, 2010 | Testing mediators of intervention effects in randomized controlled trials: An evaluation of three depression prevention programs |
| 65 | Subburaman, 2019 | Effectiveness of musical toothbrush on oral debris and gingival bleeding among 6-10-year-old children: A randomized controlled trial. |
| 66 | Sun, 2025 | The impact of background music on flow, work engagement and task performance: A randomized controlled study. |
| 67 | Tachibana, 2012 | A new mother-child play activity program to decrease parenting stress and improve child cognitive abilities: A cluster randomized controlled trial. |
| 68 | Taylor, 2021 | Child-centered play therapy and social-emotional competencies of African American children: A randomized controlled trial |
| 69 | Tobias da Silva, 2017 | Influence of therapeutic play on the anxiety of hospitalized school-age children: clinical trial. |
| 70 | Tol, 2008 | School-based mental health intervention for children affected by political violence in Indonesia - A cluster randomized trial |
| 71 | Vogl, 2009 | A computerized harm minimization prevention program for alcohol misuse and related harms: randomized controlled trial |
| 72 | WC Su, 2025 | Effects of movement and sedentary play interventions on executive functioning and their relationships with sensory, repetitive, and negative behaviors of children with ASD - a pilot RCT |
| 73 | Williams, 2018 | Improving community stroke preparedness in the HHS (Hip-Hop Stroke) randomized clinical trial |
| 74 | Wong, 2018 | Effects of therapeutic play on children undergoing cast-removal procedures: A randomised controlled trial. |
| 75 | Wright, Kingsley, Cooper, Biggs, Bursnall, H.-I. Wang,, 2023 | Play brick therapy to aid the social skills of children and young people with autism spectrum disorder: The I-SOCIALISE cluster RCT. |
| 76 | Wright, Kingsley, Cooper, Biggs, Bursnall, H.-I.- Wang,, 2023 | I-SOCIALISE: Results from a cluster randomised controlled trial investigating the social competence and isolation of children with autism taking part in LEGO ® based therapy ('Play Brick Therapy') clubs in school environments. |
| 77 | Wright, 2025 | Autism Spectrum Social Stories in Schools Trial 2 (ASSSIST-2): A pragmatic randomised controlled trial of the Social Stories intervention to address the social and emotional health of autistic children in UK primary schools. |
| 78 | Youth Leadership Council, 2020 | Pilot study of a multilevel mobile health app for substance use, sexual risk behaviors, and testing for sexually transmitted infections and hiv among youth: randomized controlled trial |
| 79 | Zarifsaniey, 2022 | Promoting self-management behaviors in adolescents with type 1 diabetes, using digital storytelling: A pilot randomized controlled trial |
| 80 | Zhang, 2024 | Recreational gymnastics exercise of moderate intensity enhances executive function in Chinese preschoolers: A randomized controlled trial |
| 81 | Zieger, 2013 | A prospective randomised control study: reduction of children's pain expectation using a picture book during blood withdrawal |
| Wrong comparator n=2 | | |
| 1 | Lin, 2024 | Effects of broadband music and audible band music on relaxation states and cognitive function in young adults: A randomized controlled trial |
| 2 | Matheson, 2020 | The effectiveness of brief animated films as a scalable micro-intervention to improve children's body image: A randomised controlled trial |
| Did not include targeted outcomes n=76 | | |
| 1 | Agley, 2021 | Effects of ACT Out! Social issue theater on social-emotional competence and bullying in youth and adolescents: cluster randomized controlled trial |
| 2 | Akard, Dietrich, Friedman, Gerhardt,, 2021 | Improved parent-child communication following a RCT evaluating a legacy intervention for children with advanced cancer |
| 3 | Akbari, 2023 | Comparing effects of massage therapy and music on chemotherapy-induced nausea and vomiting in cancer children: A randomized control trial. |
| 4 | Aléman, 2017 | The effects of musical training on child development: A randomized trial of El Sistema in Venezuela |
| 5 | Anjos, 2018 | The influence of educational dance on the motor development of children. |
| 6 | Antonson, 2018 | Upper secondary school students' compliance with two Internet-based self-help programmes: A randomised controlled trial |
| 7 | Baker, 2006 | The effect of music therapy services on classroom behaviours of newly arrived refugee students in Australia—a pilot study |
| 8 | Barry, 2010 | Music therapy CD Creation for initial pediatric radiation therapy: A mixed methods analysis |
| 9 | Batini, 2025 | Shared reading aloud fosters intelligence: Three cluster-randomized control trials in elementary and middle school |
| 10 | Bolduc, 2021 | The impact of music training on inhibition control, phonological processing, and motor skills in kindergarteners: A randomized control trial |
| 11 | Bollimbala, 2019 | Impact of acute physical activity on children's divergent and convergent thinking: The mediating role of a low body mass index |
| 12 | Chan, 2019 | Using digital storytelling to facilitate critical thinking disposition in youth civic engagement: A randomized control trial |
| 13 | Chapman, 2001 | The effectiveness of art therapy interventions in reducing Post Traumatic Stress Disorder (PTSD) symptoms in pediatric trauma patients |
| 14 | Chen, 2021 | Effects of a group music-based intervention in improving attachment and psychosocial adaptation in adolescents with parental attachment insecurity: A randomized trial |
| 15 | Cibrian, 2020 | Supporting coordination of children with ASD using neurological music therapy: A pilot randomized control trial comparing an elastic touch-display with tambourines |
| 16 | Cogo-Moreira, 2013 | Effectiveness of music education for the improvement of reading skills and academic achievement in young poor readers: A pragmatic cluster-randomized, controlled clinical trial |
| 17 | Colegrove, 2018 | Pilot randomized controlled trial of Tuning Relationships with Music: Intervention for parents with a trauma history and their adolescent |
| 18 | Colwell, 2005 | The effect of composition (art or music) on the self-concept of hospitalized children |
| 19 | Corbett, 2016 | Improvement in social competence using a randomized trial of a theatre intervention for children with autism spectrum disorder |
| 20 | Corbett, 2019 | Treatment effects in social cognition and behavior following a theater-based intervention for youth with autism |
| 21 | Duberg, 2013 | Influencing self-rated health among adolescent girls with dance intervention: A randomized controlled trial. |
| 22 | Duberg, 2020 | The effects of a dance intervention on somatic symptoms and emotional distress in adolescent girls: A randomized controlled trial |
| 23 | Erdem, 2024 | The effect of finger puppet show on the level of children’s pain and fear during the incision suturation in paediatric emergency service: A randomized controlled trial. |
| 24 | Flaugnacco, 2015 | Music training increases phonological awareness and reading skills in developmental dyslexia: A randomized control trial |
| 25 | Fong, 2022 | The effects of internet-based storytelling programs (amazing adventure against stigma) in reducing mental illness stigma with mediation by interactivity and stigma content: randomized controlled trial |
| 26 | Gattino, 2011 | Effects of relational music therapy on communication of children with autism: A randomized controlled study |
| 27 | Gelernter, 2016 | Effect of auditory guided imagery on glucose levels and on glycemic control in children with type 1 diabetes mellitus |
| 28 | Golitaleb, 2023 | Comparing the effect of music and puzzle-solving on anxiety before surgery in children: A randomized clinical trial |
| 29 | Guo, 2018 | Improved digit span in children after a 6-week intervention of playing a musical instrument: An exploratory randomized controlled trial |
| 30 | Hanrahan, 2020 | A pilot randomised control trial of digitally-mediated social stories for children on the autism spectrum |
| 31 | Harris, 2022 | School-based self-management intervention using theatre to improve asthma control in adolescents: A pilot cluster-randomised controlled trial |
| 32 | J Koenig, 2013 | Music listening has no positive or negative effects on sleep quality of normal sleepers: Results of a randomized controlled trial |
| 33 | Jacobsen, 2014 | Effects of a dyadic music therapy intervention on parent-child interaction, parent stress, and parent-child relationship in families with emotionally neglected children: A randomized controlled trial |
| 34 | Jamalpoor, 2022 | Effect of narration and painting methods on the self-concept of children with thalassemia major before and three months after intervention: A randomised clinical trial |
| 35 | Joffe, 2019 | Improving storytelling and vocabulary in secondary school students with language disorder: A randomized controlled trial |
| 36 | Jones, 2013 | A randomized controlled trial of soap opera videos streamed to smartphones to reduce risk of sexually transmitted human immunodeficiency virus (HIV) in young urban African American women |
| 37 | Kevers, 2022 | The effect of a school-based creative expression program on immigrant and refugee children's mental health and classroom social relationships: A cluster randomized trial in elementary school |
| 38 | Khemamuttanak, 2021 | Manorah training alleviates median neural tension and improves physical fitness in sedentary young adults: A randomized control trial |
| 39 | Kim, 2017 | Effects of community-based group music therapy for children exposed to ongoing child maltreatment & poverty in South Korea: A block randomized controlled trial |
| 40 | Kristjánsdóttir, 2011 | Randomized clinical trial of musical distraction with and without headphones for adolescents' immunization pain |
| 41 | Kurudirek, 2021 | Effects of therapeutic clowning on pain and anxiety during venous blood sampling in Turkey: Randomised controlled trial |
| 42 | Küçükkaya, 2024 | The effect of listening to music and drawing on coping with dysmenorrhea complaints in nursing students: randomized controlled trial |
| 43 | LaGasse, 2014 | Effects of a music therapy group intervention on enhancing social skills in children with autism |
| 44 | Lewis-Smith, 2023 | A short-form drama series created for the digital media environment: A randomised controlled trial exploring effects on girls' body satisfaction, acceptance of appearance diversity, and appearance-related internalised racism |
| 45 | Lin, 2014 | Mozart K.448 listening decreased seizure recurrence and epileptiform discharges in children with first unprovoked seizures: A randomized controlled study |
| 46 | Lyshak-Stelzer, 2007 | Art Therapy for adolescents with posttraumatic stress disorder symptoms: A pilot study |
| 47 | Macri’, 2024 | The effect of test anxiety on high-stakes exams: Evidence from an RCT using biomarkers |
| 48 | Marrades-Caballero, 2018 | Neurologic music therapy in upper-limb rehabilitation in children with severe bilateral cerebral palsy: A randomized controlled trial |
| 49 | Montero-Ruiz, 2020 | Effects of music therapy as an adjunct to chest physiotherapy in children with cystic fibrosis: A randomized controlled trial |
| 50 | Obersnel, 2023 | Active production of music as distraction for venipuncture in children and adolescents: A randomized clinical trial |
| 51 | Pakiş Çetin, 2024 | Comparative effectiveness of Hegu point ice massage versus music for dysmenorrhea-related pain and comfort levels: A randomized controlled trial. |
| 52 | Palvan, 2021 | The effect of exchanging drawings with peers on the happiness of children with cancer, aged 7-11 years: A clinical trial |
| 53 | Philips, 2019 | Didgeridoo sound meditation for stress reduction and mood enhancement in undergraduates: A randomized controlled trial. |
| 54 | Rabeyron, 2020 | A randomized controlled trial of 25 sessions comparing music therapy and music listening for children with autism spectrum disorder |
| 55 | Raja, 2025 | Comparative evaluation of music and movement therapy and visual pedagogy as interventional tools in promoting oral health in children with autism spectrum disorder: A randomized controlled trial |
| 56 | Regev, 2005 | The psychological benefits of artwork: The case of children with learning disorders |
| 57 | Robb, 2014 | Randomized clinical trial of therapeutic music video intervention for resilience outcomes in adolescents/young adults undergoing hematopoietic stem cell transplant |
| 58 | Rousseau, 2014 | A cluster randomized-controlled trial of a classroom-based drama workshop program to improve mental health outcomes among immigrant and refugee youth in special classes |
| 59 | Rudd, 2021 | Comparing the efficacy (RCT) of learning a dance choreography and practicing creative dance on improving executive functions and motor competence in 6-7 years old children |
| 60 | Sandberg, 2020 | Dance intervention for adolescent girls: Effects on daytime tiredness, alertness and school satisfaction. A randomized controlled trial |
| 61 | Sayar, 2025 | The effect of health belief model-based creative drama education on health locus of control and smoking perception in adolescents: A randomized controlled trial |
| 62 | Suleman, 2024 | Comparing the use of colored Trace Images in Kids' Books with passive distractions to decrease pain and fear during cannulation in children: A randomized clinical trial |
| 63 | Suleman, 2022 | Effectiveness of art-based distraction on reducing pediatric patients' pain and anxiety during venipuncture: A randomized controlled trial |
| 64 | Suleman, 2022 | Effectiveness of art-based distraction in reducing pain and anxiety of hospitalized children during cannulation procedure: A randomized controlled trial |
| 65 | Suresh, 2015 | The effect of audio therapy to treat postoperative pain in children undergoing major surgery: A randomized controlled trial |
| 66 | Takahashi, 2023 | The effectiveness of dance movement therapy for individuals with down syndrome: A pilot randomised controlled trial |
| 67 | Teixeira-Machado, 2022 | A pilot randomized controlled clinical trial of dance practice for functionality in autistic children and adolescent with all levels of need support |
| 68 | Tuominen, 2017 | The effect of a movement-to-music video program on the objectively measured sedentary time and physical activity of preschool-aged children and their mothers: A randomized controlled trial |
| 69 | Tuominen, 2020 | Relationship between mothers' enjoyment and sedentary behavior and physical activity of mother-child dyads using a movement-to-music video program: A secondary analysis of a randomized controlled trial |
| 70 | Uhlig, 2018 | "Being a bully isn't very cool...": Rap & Sing Music Therapy for enhanced emotional self-regulation in an adolescent school setting - a randomized controlled trial |
| 71 | Uhlig, 2019 | Rap & Sing Music Therapy and sleep in adolescents: A single-blind cluster randomized controlled trial |
| 72 | Su, 2025 | Effects of creative movement, general movement, or seated play interventions on motor performance in children with autism spectrum disorder: A pilot randomized controlled trial |
| 73 | Wang, 2013 | A home-based program using patterned sensory enhancement improves resistance exercise effects for children with cerebral palsy: A randomized controlled trial |
| 74 | Yousefi, 2024 | Hypnotherapy, relaxation, and music therapy in pediatric cancer pain management: A clinical trial comparison |
| 75 | Yum, 2024 | Music therapy improves engagement and initiation for autistic children with mild intellectual disabilities: A randomized controlled study |
| 76 | Zhang, 2023 | A randomized control trial establishing the effectiveness of using interactive television-based art, music, and poetry therapies for treating the post-traumatic stress disorder of children exposed to traumatic events |
| Wrong study design n=7 | | |
| 1 | Hill, 2016 | Improving the short-term affect of grieving children through art |
| 2 | Hilliard, 2007 | The effects of Orff-based music therapy and social work groups on childhood grief symptoms and behaviors |
| 3 | Jones, 2012 | Streaming weekly soap opera video episodes to smartphones in a randomized controlled trial to reduce hiv risk in young urban african american/black women |
| 4 | DeMott, 2017 | A controlled early group intervention study for unaccompanied minors: Can Expressive Arts alleviate symptoms of trauma and enhance life satisfaction? |
| 5 | Moula, 2021 | "I didn't know I have the capacity to be creative": Children's experiences of how creativity promoted their sense of well-being. A pilot randomised controlled study in school arts therapies |
| 6 | Oppici, 2020 | Efficacy of a 7-week dance (RCT) PE curriculum with different teaching pedagogies and levels of cognitive challenge to improve working memory capacity and motor competence in 8-10 years old children |
| 7 | Robb, 2008 | Randomized controlled trial of the active music engagement (AME) intervention on children with cancer |
| Wrong article type n=40 | | |
| 1 | Akard, 2020 | Parent-child communication outcomes of a RCT testing a legacy intervention for children with advanced cancer (GP703) |
| 2 | Babaei, 2021 | Painting therapy versus anxiolytic premedication to reduce preoperative anxiety levels in children undergoing tonsillectomy: A randomized controlled trial |
| 3 | Barrera, 2013 | A randomized control trial to evaluate the efficacy of a group social skills intervention for childhood survivors of brain tumours |
| 4 | Barrera, 2014 | Reduction of anxiety levels in parents and siblings of children with cancer after sibling participation in a psychosocial group intervention: A randomized controlled trial |
| 5 | Bay, 2020 | 602 Effect of music in pediatric endoscopic examinations: A randomized controlled trial |
| 6 | Bittman, 2009 | Creative musical expression as a catalyst for quality-of-life improvement in inner-city adolescents placed in a court-referred residential treatment program. |
| 7 | Bonnot, 2015 | Simple blind randomized controlled trial of music therapy versus music listening in patients with autism spectrum disorders regarding efficiency in behaviour and communication disorders. Preliminary results |
| 8 | Brandes, 2010 | Receptive music therapy for the treatment of depression: A proof-of-concept study and prospective controlled clinical trial of efficacy. |
| 9 | Capitulo, 2015 | Music therapy to reduce pain and anxiety in children with cancer undergoing lumbar puncture: A randomized clinical trial |
| 10 | Carlon, 2014 | Aerobic training for young people with cerebral palsy in specialist schools: A pilot randomised controlled trial |
| 11 | Doherty, 2022 | Emotional awareness and expression therapy and mindfulness meditation training for college students with anxiety and somatic symptoms: A randomized controlled trial |
| 12 | Festini, 2014 | Effectiveness of a new method to reduce preoperative anxiety in children: Randomised controlled trial |
| 13 | G Park, 2016 | Randomized single-blinded clinical trial on effects of nursery songs for infants and young children's anxiety before and during head CT |
| 14 | G. Park, 2016 | Randomized single-blinded clinical trial on effects of nursery songs for infants and young children's anxiety before and during head computed tomography |
| 15 | Gelernter, 2014 | A blinded randomized trial: The effect of guided imagery in children with type 1 diabetes mellitus on glucose levels and on glycemic control |
| 16 | Geretsegger, 2014 | TIME-A-an international RCT on the effectiveness of music therapy in ASD |
| 17 | Gozin, 2023 | Erratum: Correction to: Audio-visual storytelling for reducing dental anxiety in Iranian children: A randomized controlled trial |
| 18 | Grace Park, 2016 | Randomized single-blinded clinical trial on effects of nursery songs for infants and young children's anxiety before and during head CT...Ayubi E. Effects of nursery songs for infants and young children's anxiety: A reporting mistake of effect measure of |
| 19 | Groessing, 2023 | Design and implementation of a replication study: The Music for Autism (M4A) binational assessor-blinded randomised crossover trial |
| 20 | Herlekar, 2013 | Effect of classical instrumental music on perceived stress, a comparison between Indian and Malaysian 1st yr medical girls-A randomised control trial |
| 21 | Herlekar, 2018 | Reworking periodic blood pressure response to successive divided attention tests when performed with instrumental background music - A randomised controlled trial |
| 22 | Hoffman, 2019 | A pilot cluster randomized, controlled trial of a clerkship-based visual arts curriculum to combat bias and promote well-being |
| 23 | Jubin, 2017 | A randomized controlled study in young adults to compare changes in creativity after Quadrato motor training versus simple motor training |
| 24 | Klim-Conforti, 2021 | 31.1 The impact of a Harry Potter-based CBT skills curriculum on suicidality and well-being in middle schoolers: A randomized controlled trial |
| 25 | Kocherov, 2015 | The influence of the use medical clowns to the reduction of preoperative anxiety, postoperative pain and medical costs in children undergoing outpatient penile surgery: A randomized controlled trial |
| 26 | Koenig, 2012 | Music therapy for chronic headache in adolescents - A randomized controlled trial |
| 27 | Lai, 2022 | Home-based telehealth movement-to-music increases physical activity participation among adolescents with cerebral palsy: Pilot RCT |
| 28 | Lance, 2013 | Can 16 and pregnant affect attitudes towards teen pregnancy among young women? A randomized controlled trial |
| 29 | Leask Capitulo, 2015 | Toward evidence-based practice. Music therapy to reduce pain and anxiety in children with cancer undergoing lumbar puncture: A randomized clinical trial. |
| 30 | Li, 2018 | A pilot randomized controlled trial of using musical training in promoting psychological well-being and quality of life in childhood brain tumours survivors |
| 31 | Li, 2018 | Effectiveness of musical training in reducing depressive symptoms and improving quality of life in childhood brain tumours survivors: A randomized controlled trial |
| 32 | McCann, 2012 | Bibliotherapy for first-time primary caregivers of family members with first-episode psychosis: A randomized controlled trial |
| 33 | McCann, 2013 | Bibliotherapy for primary carers of young people with a first episode of psychosis: Randomised controlled trial |
| 34 | Moon, 2010 | Effects of therapeutic music play based on theory of mind for children with Asperger syndrome - self-cognition, self-efficacy, and self-esteem |
| 35 | Nabavi, 2021 | Investigating the effect of expressive writing on level of sexual dysfunction in Iranian women with multiple sclerosis: A randomized controlled trial |
| 36 | Raja, 2024 | Comparative evaluation of music therapy and visual pedagogy as interventional tools in promoting oral health in children with autism spectrum disorder - Randomized controlled trial. |
| 37 | Swedberg Yinger, 2013 | Music therapy as procedural support for young children undergoing immunizations: A randomized controlled study. |
| 38 | Turry, 2018 | Response to effects of improvisational music therapy vs. enhanced standard care on symptom severity among children with autism spectrum disorder: The TIME-A randomized clinical trial |
| 39 | Wang, 2012 | A randomized controlled trial of neurologic music therapy during a functional strengthening program for children with cerebral palsy |
| 40 | Williams, 2017 | RCT data on the effect of stroke education on children's knowledge and self-efficacy |
| Subset of participants already included in another study n=1 | | |
| 1 | Moula, 2023 | Child-focused outcome and process evaluation of a school-based art therapy intervention: A pilot randomised controlled study |

## Supplementary Table 8. List of eligible studies from top-up search on 30/4/2026 (not included in this review)

|  | **First author, year** | **Title** |
| --- | --- | --- |
| 1 | Zhu, 2026 | The mediating effects of digital music therapy on depressive symptoms in youth during the COVID‐19 pandemic: A randomized controlled trial. |
| 2 | Köksal, 2025 | Drawing and mutual storytelling to alleviate anxiety and improve emotional well-being in children in PICU: A randomized controlled trial. |
| 3 | Akay, 2025 | Mandala coloring reduces anxiety and pain in circumcised children: A randomized controlled trial. |
| 4 | Kuppusamy, 2025 | Effectiveness of musical drama, music listening, and tell‐show‐do techniques on reducing dental anxiety among orphaned children aged 12–15 years—a randomized controlled trial. |
| 5 | Gocen, 2025 | The effect of therapeutic artistic activities on anxiety and psychological well-being in adolescents with type 1 diabetes: A randomized controlled study. |
| 6 | Semerci Şahin, 2025 | The effect of a musical starry sky projector on fatigue, sleep, and quality of life in pediatric oncology patients: A randomized Controlled Trial. |
| 7 | Meerza, 2025 | The effectiveness of an interactive toy as an active distraction compared with a passive distraction in alleviating dental anxiety and pain while administration of local anaesthesia in children: A randomised clinical Trial |
| 8 | Hakim, 2025 | The effect of storytelling on the social anxiety of hospitalized children with burns: A clinical trial study |
| 9 | Lam, 2026 | Evaluating the sound magnetic balance intervention in a randomized controlled trial: Effects on psychological distress, somatic pain, and physiological arousal. |
| 10 | Kaş Alay, 2026 | Effect of therapeutic storytelling on intramuscular injection-related pain, anxiety, and fear in children: A randomized controlled trial. |
| 11 | Park, 2026 | Preparing for future pandemics: enhancing serotonin activation and interpersonal warmth through music therapy to aid in adolescent depression recovery post-COVID-19: A randomized controlled trial. |
| 12 | Agres, 2025 | The impact of performing arts on mental health, social connection, and creativity in university students: A randomised controlled trial. |
| 13 | Wang, 2025 | Mindfulness-based music therapy for mental health in senior college students: A randomized controlled trial. |
| 14 | Unal, 2025 | Virtual reality and music effects on skin test discomfort: A randomized controlled trial. |
| 15 | Wu, 2025 | Alleviating adolescent stress, anxiety, and fear through music and sports games during the COVID-19 pandemic: A randomized controlled trial. |
| 16 | Yang, 2025 | Intervention effect of CBT-based poetry therapy on promoting positive psychology: Randomized controlled trials on Chinese adolescents. |
| 17 | Lin, 2025 | Effectiveness of a school-based music intervention program on the mental health of middle school adolescent: A cluster randomized controlled trial. |
| 18 | Liu, 2026 | Combining dance movement therapy and pharmacotherapy in adolescents with major depressive disorder: A randomized controlled study |
